# Pathway-Specific Engineered Mouse Allograft Models Functionally Recapitulate Human Serous Epithelial Ovarian Cancer

**DOI:** 10.1371/journal.pone.0095649

**Published:** 2014-04-18

**Authors:** Ludmila Szabova, Sujata Bupp, Muhaymin Kamal, Deborah B. Householder, Lidia Hernandez, Jerome J. Schlomer, Maureen L. Baran, Ming Yi, Robert M. Stephens, Christina M. Annunziata, Philip L. Martin, Terry A. Van Dyke, Zoe Weaver Ohler, Simone Difilippantonio

**Affiliations:** 1 Center for Advanced Preclinical Research, Leidos Biomedical Research, Inc., Frederick National Laboratory for Cancer Research, Frederick, Maryland, United States of America; 2 Medical Oncology Branch, National Cancer Institute, Bethesda, Maryland, United States of America; 3 Advanced Biomedical Computing Center, Leidos Biomedical Research, Inc., Frederick National Laboratory for Cancer Research, Frederick, Maryland, United States of America; 4 Mouse Cancer Genetics Program, Frederick National Laboratory for Cancer Research, Frederick, Maryland, United States of America; National Cancer Center, Japan

## Abstract

The high mortality rate from ovarian cancers can be attributed to late-stage diagnosis and lack of effective treatment. Despite enormous effort to develop better targeted therapies, platinum-based chemotherapy still remains the standard of care for ovarian cancer patients, and resistance occurs at a high rate. One of the rate limiting factors for translation of new drug discoveries into clinical treatments has been the lack of suitable preclinical cancer models with high predictive value. We previously generated genetically engineered mouse (GEM) models based on perturbation of *Tp53* and *Rb* with or without *Brca1* or *Brca2* that develop serous epithelial ovarian cancer (SEOC) closely resembling the human disease on histologic and molecular levels. Here, we describe an adaptation of these GEM models to orthotopic allografts that uniformly develop tumors with short latency and are ideally suited for routine preclinical studies. Ovarian tumors deficient in *Brca1* respond to treatment with cisplatin and olaparib, a PARP inhibitor, whereas *Brca1*-wild type tumors are non-responsive to treatment, recapitulating the relative sensitivities observed in patients. These mouse models provide the opportunity for evaluation of effective therapeutics, including prediction of differential responses in *Brca1*-wild type and *Brca1*–deficient tumors and development of relevant biomarkers.

## Introduction

Ovarian cancer is the second most common gynecologic cancer and the most frequent cause of gynecologic cancer-related deaths in the USA [1], with over 50% presenting as serous epithelial ovarian cancer (SEOC). Most advanced SEOCs have spread beyond the ovary at the time of diagnosis, and their management involves surgical de-bulking, followed by chemotherapy with a combination of platinum and taxane drugs [2], [3]. Despite initially high response rates, most patients relapse with a median progression-free survival of 18 months [4], making the search for new therapeutics imperative.

Over the last several years much progress has been made in identifying hallmark genetic lesions associated with SEOC. The Cancer Genome Atlas (TCGA) study of a large high grade SEOC cohort revealed that mutations in *TP53* predominated, occurring in at least 96% of tumors [5]. Alterations in the RB network were observed in 67% of cases, and about 20% of tumors had germ line or somatic mutations in *BRCA1/2* with an additional 11% having lost BRCA1 expression through epigenetic silencing [5].

BRCA1 and BRCA2 play important roles in homologous recombination (HR), and loss of either protein leads to deficiency in double strand DNA break (DSB) repair [6]. In HR-deficient cells, DSBs are repaired by error-prone mechanisms, which can lead to genomic instability. Chemical inhibition of single strand (ss) DNA break repair in HR-deficient cells can lead to synthetic lethality due to the concurrent absence of two DNA repair pathways [7], [8]. In the clinic, poly(ADP)-ribose polymerase inhibitors (PARPi) are undergoing evaluation as a method of exploiting synthetic lethality for therapeutic intervention. PARP-1 is required for identification of ssDNA breaks (SSB) and recruitment of repair proteins into damaged sites [9]. Inhibition of this process in BRCA-deficient cells leads to the accumulation of DSBs, due to collapsed replication forks, and ultimately to cell death [10]–[12]. Based on these findings, PARPi have been tested in clinical trials as single or combination therapies in patients with advanced solid tumors [13]–[15]. Recently, the orally active PARPi, olaparib (AZD2281), showed clinical antitumor activity in BRCA-associated ovarian and breast cancers [15]–[19].

Thus far, clinical trials of new therapeutic agents have relied on preclinical studies performed in cell lines and immunocompromised cell line xenograft models. Subsequent high failure rates in human trials suggest a need for preclinical models with better predictive value [20]. Genetically engineered mouse models (GEMMs) have important advantages over cell line xenograft models, in that the tumors are driven by defined genetic events and arise due to the accumulation of additional stochastic events within the organ of interest and are, therefore, subject to relevant microenvironment and host cell responses. Several epithelial ovarian cancer GEMMs that develop tumors resembling human disease have been reported [21]–[25]; however, these models have not been tailored for efficient use in preclinical studies, and few recapitulate the full range of genetic and biological features of human SEOC.

Previously, we reported GEMMs that develop ovarian cancers with the genetic and biological characteristics of human SEOCs [26]. Targeted to the ovarian surface epithelium, abrogation of RB tumor suppression (TS) initiated disease, and *Tp53* aberration (missense mutation or deletion) facilitated disease progression to aggressive peritoneal dissemination and metastasis. Ovarian tumors from these models, including those deficient in either *Brca1* or *Brca2*, represent all 4 transcriptional subclasses of human SEOC. Here, we utilize these models to create orthotopic immunocompetent transplant models, and to generate synchronized cohorts of mice suitable for preclinical studies. To determine whether these models are tractable for use in routine efficacy studies and if their therapeutic responses reflect observed outcomes in human trials, we performed single or combination treatment with standard platinum chemotherapy and olaparib. As observed in patients, treatment response was dependent on *Brca1* status, thereby demonstrating the utility of these models in evaluating the potential efficacy of novel therapeutics for ovarian cancer.

## Material and Methods

### Experimental animals

All animal experiments were performed in accordance to animal study protocol approved by the Institutional Animal Care and Use Committee of Frederick National Laboratory for Cancer Research and the National Institutes of Health Guide for the Care and Use of Laboratory Animals. FNLCR is accredited by AAALAC International and follows the Public Health Service Policy for the Care and Use of Laboratory Animals. Animal care was provided in accordance with the procedures outlined in the “Guide for Care and Use of Laboratory Animals” (National Research Council; 1996; National Academy Press; Washington, D.C.). Animals were kept in a barrier facility at FNLCR under HEPA filtration in micro isolator cages and fed with autoclaved laboratory rodent diet (LabDiet, St. Louis, MO). All animal procedures were performed under anesthesia using inhalation of 1–2.5% isoflurane. The endpoint for mice was change in general health, specifically >20% body weight loss, inability to eat, drink, or ambulate, hunched posture, difficulty breathing or signs of hypothermia as well as signs of ascites with abdominal distension and palpable tumor of approximately 2 cm size. Animals that exhibited clinical signs, solid tumor growth of 2cm, or became moribund before maximum ascites expansion (approximate doubling of the width of the abdomen) were promptly euthanized by CO2 inhalation.

Detailed mouse strain information and generation of adenovirally induced SEOC models have been described previously [26]. Beige nude [Cr:NIH-bg-nu-Xid], athymic nude [Athymic NCr-nu/nu] and FVB [FVB/NCr] female mice were obtained from the Animal Production Program, FNLCR or from Jackson Laboratories [FVB/NJ].

### Orthotopic tumor transplantation

To perform orthotopic tumor transplantation, the lumbar region of FVB females (5–8 weeks old) was shaved, animals were anesthetized and the skin was aseptically prepared for surgery. A lateral longitudinal skin incision was made in the flank region overlying the right ovary and another smaller incision was made in the peritoneum. The exposed ovary, with the surrounding fat pad, was exteriorized and a small donor tumor fragment (about 2 mm) was inserted under the bursa through a small surgical tear. The ovaries were replaced back into the abdominal cavity, the peritoneum was sutured and the skin closed with metal clips.

### Cell cultures

Human ovarian carcinoma cell line HeyA8 was a gift from Dr. Elise Kohn, NCI, and was originally described in [27]; lines 1A9 and cisplatin-resistant 1A9CP80 were gifts from Dr. Tito Fojo (NCI), and were derived as in [28], [29]; *BRCA1* mutant line UWB1.289 was obtained from ATCC (Manassas, VA). All ovarian lines were cultured in RPMI (Life Technologies, Grand Island, NY) with 10% fetal bovine serum (Hyclone, Pittsburg, PA) and standard antibiotics; 1A9CP80 cell medium was supplemented with 80 mM cisplatin (Tocris, Bristol,UK). A detailed procedure for establishment of murine ovarian cancer cell lines is provided in Data S1.

### *In vitro* cytotoxicity assays

Viability of the attached ovarian cancer cells was assessed using XTT as described [30]. Briefly, cells were seeded in 96-well plates at a density of 1–2,000 cells/50 µl/well and incubated for 24 h. Cisplatin (Tocris, Bristol,UK) and olaparib (AZD2281, Selleck Chemicals, Houston, TX) were prepared in dimethyl sulfoxide (DMSO) stocks and applied to plated cells from freshly prepared working solutions serially diluted in medium. Cells were replenished with fresh drugs after 3 days and the viability was determined after 7 days by incubating cultures with XTT and measuring the absorbance using Tecan F200 plate reader (Research Triangle Park, NC). Cell density in treated wells was expressed as percentage of control. Experiments included triplicate samples and were repeated at least three times. IC50 values (concentrations producing loss of viability in 50% of cells) were calculated by linear regression.

### Preclinical studies in mice

For *in vivo* studies, cisplatin (Tocris, Bristol, UK) was reconstituted in sterile 0.9% saline solution and injected intraperitoneally, and olaparib (AZD2281, Selleck Chemicals, Houston, TX) was administered orally in vehicle [PBS containing 10% DMSO (Sigma, St. Luis, MO) and 10% (2-Hydroxypropyl)-cyclodextrin (Sigma, St. Luis, MO)]. For the pharmacodynamic studies mice with established orthotopic ovarian tumors (∼0.5 cm in diameter) were treated with: 1) cisplatin (5 mg/kg), n = 3; 2; 3 and 3 for line 29255; 32233; 39877 and 30200; respectively, 2) olaparib (50 mg/kg), n = 1; 3; 2 and 3 for line 29255; 32233; 39877 and 30200; respectively 3) cisplatin (5 mg/kg) followed by olaparib (50 mg/kg) 1 hr later, n = 3; 2; 2 and 2 for line 29255; 32233; 39877 and 30200; respectively or 4) vehicle, n = 2; 2; 2 and 1 for line 29255; 32233; 39877 and 30200; respectively, on day 1. Groups 2 and 4 received another dose of olaparib and group 4 another dose of vehicle 24 hrs later. All animals were euthanized on day 2, two hours after last dosing. Tumor tissues were collected and PAR levels were measured using the HT PARP *in vivo* Pharmacodynamic Assay II (Trevigen, Gaithersburg, MD). Tumor lysates were prepared according to manufacturer's instructions and 2 µg of total proteins were analyzed.

For efficacy and long term treatment studies animals with palpable tumors (∼0.5 cm in diameter) were imaged and randomized into 4 treatment groups, as described above, based on tumor volume to achieve approximately same distribution of volumes in all groups. Cisplatin was administered intraperitoneally once a week. Olaparib and vehicle were administered orally for 5 consecutive days per week. Animals in efficacy studies were treated for 2 or 3 weeks, imaged to determine changes in tumor volume and euthanized 2 hours post olaparib/vehicle dosing. Animals in long-term dosing study were treated for up to 10 weeks and imaged biweekly using ultrasound (US) to determine the tumor volumes. They were monitored after cessation of treatment for tumor development and were euthanized once moribund due to tumor burden. Tumor tissue was collected for histological analysis and immunohistochemistry (IHC).

### Gene expression profiling

Gene expression profiling and comparison of murine and human datasets was performed as described previously [26]. Data for gene expression analysis of murine primary and derived orthotopic SEOC tumors are publicly available at Gene Expression Omnibus database under accession number GSE51927.

Data S1 contain material and methods for establishment of murine primary tumor cell lines, cell implantations, imaging and tumor volume measurements, tissue collection, pathological and IHC analysis, quantitative analysis of IHC stains, immunofluorescent staining of human and murine cells and quantitative PCR analysis of *Brca1* status.

## Results

### Transplant models retain biological and genetic features of primary SEOCs

GEM SEOC models were induced by injection of an adenoviral Cre vector into the ovarian bursa of mice harboring various combinations of Cre-dependent alleles. As described previously [26], when RB-TS function was inactivated via expression of *TgK18_T121_* allele and was combined with *Tp53* deletion or missense mutation (*p53^R172H^*), cancers evolved over the course of 8–12 months to produce high grade metastatic disease. Loss of *Brca1* or *2* combined with RB-TS inactivation did not result in disease progression beyond stage I without concurrent Tp53 aberration, and did not noticeably influence the biology of disease progression when combined with perturbation of *Rb* and *Tp53*. However, *Brca1* or *Brca2* status clearly impacts certain therapeutic outcomes in humans; therefore it was important to maintain models both with and without wild type *Brca1* for preclinical studies. The *de novo* SEOC models are excellent for understanding disease etiology and for biomarker discovery, however, they exhibit a long latency to advanced disease, making the timing of cohort production for preclinical studies challenging. Additionally, due to presence of the p53^R172H^ allele (a null allele prior to recombination), SEOC GEM models may develop tumors outside of the ovary such as lymphomas and sarcomas [26], reducing the predictable cohort size. These features render the models sub-optimal for preclinical therapeutic studies.

**Figure 1:**
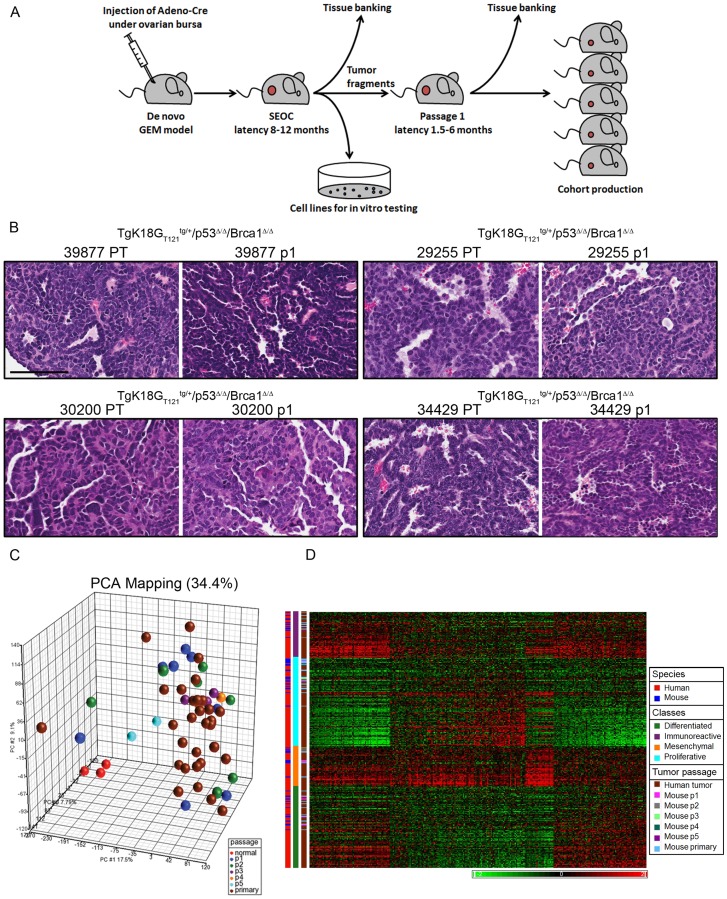
Development and characterization of orthotopic models for SEOC. A, Allograft models of SEOC were generated by transplantation of an ovarian tumor fragments from the *de novo* models of SEOC under the bursae of syngeneic immunocompetent mice. Primary ovarian carcinoma cell lines were generated simultaneously. The latency for tumor development in orthotopic models shortened substantially compared to the latency of the *de novo* model. B, H&E of primary tumors (PT) and corresponding passage 1 (p1) tumors from 4 different tumor lines indicating SEOC histology in PT and derived orthotopic tumor transplants. Scale bar represents 100 µm. C, Principal component analysis of normal ovarian surface epithelium, primary ovarian tumors and different passages of derived orthotopic tumors. D, Cluster analysis of merged human and mouse data using classifier gene sets showed that passaged tumors, similarly to primary tumors, represented all 4 subgroups of human SEOC originally derived from TCGA study [5].

To adapt these models to effective preclinical tools preserving SEOC stromal characteristics and immunocompetency, we optimized orthotopic transplantation of tumor fragments from primary GEM-derived SEOCs into the ovarian bursa of recipient mice (Fig. 1A). Of 35 primary tumors, 17 tumors were successfully passaged into syngeneic recipient mice (Table 1). The take rate in passage 1 varied from 20–100% among different tumor lines with marked differences in take rate between *Brca1* null (*TgK18G_T121_^tg/+^/Brca1^Δ/Δ^/p53^Δ/Δ^)* and *Brca1* wild type *(TgK18G_T121_^tg/+^/p53^Δ/Δ^)* tumors (95%±5.0 vs 64.5%±5.6, p<0.01). For both genotypes, the latency of development to terminal stage was substantially shortened from 9.95±1.29 to 2.12±0.61 months compared to the de novo model (Table 1). Subsequent *in vivo* passaging further shortened latencies and often improved take rates (Table 1). We also observed differences in latency between passaged tumors of *TgK18G_T121_^tg/+^/Brca1^Δ/Δ^/p53^Δ/Δ^* v*s TgK18G_T121_^tg/+^/p53^Δ/Δ^* genotypes in the first (P1) (2.8±0.2 months vs 3.6±0.1 months, p<0.01) and second (P2) passage (1.8±0.1 months vs 2.7±0.2 months, p<0.01). Of note, we also attempted to establish orthotopic allograft models using cells cultured from primary tumors or ascites. While such cultures could be readily established *in vitro* and proved useful for evaluation of drug cytotoxicity (see below), orthotopic implantation *in vivo* was much less successful in efficiently establishing tumors with required fidelity compared with direct transplantation of tumor tissues in syngeneic recipients (Data S1).

**Table 1:** Summary of orthotopic syngeneic tumor transplants.

		Primary	P1			P2		
Line	Genotype	Latency* (months)	Take rate (N)	Average latency* (months)	Histology (inter-tumor)	Take rate (N)	Average latency* (months)	Histology (inter-tumor)
**30200**	TgK18G_T121_^tg/+^/ Brca1^Δ/Δ^/p53^Δ/Δ^	7.3	100% (5/5)	2.7	80% SEOC, poorly differentiated papillary; 20% SEOC, papillary	100% (5/5)	2.4	60% SEOC, poorly differentiated papillary; 40% undifferentiated carcinoma
**36799**	TgK18G_T121_^tg/+^/ Brca1^Δ/Δ^/p53^Δ/Δ^	8.7	100% (6/6)	3.2	100% SEOC, papillary	87% (13/15)	2.2	77% SEOC, papillary; 23% SEOC, poorly differentiated papillary
**33641**	TgK18G_T121_^tg/+^/ Brca1^Δ/Δ^/p53^Δ/Δ^	11.1	60% (3/5)	3.4	100% SEOC, poorly differentiated papillary	90% (9/10)	1.4	67% undifferentiated carcinoma; 22% SEOC, poorly differentiated papillary; 11% SEOC, papillary
**39877**	TgK18G_T121_^tg/+^/ Brca1^Δ/Δ^/p53^Δ/Δ^	8.4	100% (4/4)	3.2	100% SEOC, papillary	100% (5/5)	1.4	100% SEOC, papillary
**33364**	TgK18G_T121_^tg/+^/ Brca1^Δ/Δ^/p53^Δ/Δ^	9.6	100% (5/5)	3.2	80% SEOC, papillary; 20% SEOC, poorly differentiated papillary	100% (23/23)	1.5	70% SEOC, papillary; 30% SEOC, poorly differentiated papillary
**39647**	TgK18G_T121_^tg/+^/ Brca1^Δ/Δ^/p53^Δ/Δ^	9.8	100% (5/5)	1.6	60% SEOC, poorly differentiated papillary; 40% undifferentiated carcinoma	100% (9/9)	1.5	100% undifferentiated carcinoma
**36685**	TgK18G_T121_^tg/+^/ Brca1^Δ/Δ^/p53^Δ/Δ^	9.8	100% (5/5)	2.4	100% SEOC, papillary	100% (13/13)	1.8	92% SEOC, papillary; 8% SEOC, poorly differentiated papillary
**39646**	TgK18G_T121_^tg/+^/ Brca1^Δ/Δ^/p53^Δ/Δ^	8.6	100% (4/4)	2.60	75% SEOC, papillary; 25% SEOC, poorly differentiated papillary	100% (5/5)	1.9	100% SEOC, papillary
**22864**	TgK18G_T121_^tg/+ ^/Brca1^Δ/Δ^/p53^R172H/Δ^	10.6	50% (2/4)	5.4	100% SEOC, papillary	54% (7/13)	1.9–5.6	71% SEOC, papillary; 29% SEOC, poorly differentiated papillary
**25604**	TgK18G_T121_^tg/+^/ Brca2^Δ/Δ^/p53^R172H/Δ^	9.4	20% (1/5)	5.9	100% SEOC, papillary	0% (0/4)	N/A	N/A
**27719**	TgK18G_T121_^tg/+^/ Brca2^Δ/Δ^/p53^R172H/Δ^	8.9	100% nudes (5/5)0% FVB (0/5)	2.8 nudes	60% SEOC, papillary; 20% SEOC, poorly differentiated papillary; 20% undifferentiated carcinoma	100% nudes (5/5) 20% FVB (1/18)	3.2 nudes 3.5 FVB	FVB: 100% SEOC, poorly differentiated papillary; NUDES: 60% SEOC, papillary; 40% SEOC, poorly differentiated papillary
**29255**	TgK18G_T121_^tg/+^/ p53^Δ/Δ^	11.7	60% (3/5)	4	33% SEOC, papillary; 33% SEOC, poorly differentiated; 33% no diagnosis available	93% (13/14)	2.2	54% SEOC, papillary; 38% SEOC, poorly differentiated papillary; 8% undifferentiated carcinoma
**34429**	TgK18G_T121_^tg/+^/ p53^Δ/Δ^	10.9	50% (2/4)	3.6	66% SEOC, papillary; 33% no diagnosis available	80% (4/5)	3.2	25% no diagnosis; 75% SEOC, papillary
**36341**	TgK18G_T121_^tg/+^/ p53^Δ/Δ^	10.3	50% (2/4)	3.95	50% undifferentiated carcinoma; 50% SEOC, poorly differentiated papillary	60% (3/5)	3	66% undifferentiated carcinoma; 33% SEOC, poorly differentiated papillary
**32233**	TgK18G_T121_^tg/+^/ p53^Δ/Δ^	11	80% (4/5)	3.1	100% SEOC, papillary	100% (4/4)	2.8	100% SEOC, papillary
**34706**	TgK18G_T121_^tg/+^/ p53^Δ/Δ^	11.9	80% (4/5)	3.7	50% undifferentiated carcinoma; 25% SEOC, papillary; 25% SEOC, poorly differentiated papillary	70% (7/10)	2.3	57% SEOC, poorly differentiated papillary; 29% SEOC, papillary; 14% undifferentiated carcinoma
**35087**	TgK18G_T121_^tg/+^/ p53^Δ/Δ^	11.2	67% (2/3)	3.5	100% SEOC, papillary	N/A	N/A	N/A

*latency of tumor development from induction or transplantation till end point.

*TgK18G_T121_^tg/+^  = * transgenic for bacterial artificial chromosome containing the mouse cytokeratin 18 gene, into which a Cre-conditional loxP-GFP-stop-loxP T_121_ cassette was inserted.

*Brca1^Δ/Δ^  = * deletion mutant for *Brca1* gene.

*Brca2^Δ/Δ^  = * deletion mutant for *Brca2* gene.

*p53^Δ/Δ^*  =  deletion mutant for p53 gene.

*p53^R172H/Δ^*  = point mutation and deletion mutant for *p53* gene.

To determine whether significant changes in tumor phenotype were associated with increased passage number, primary and orthotopic tumor histopathologies were assessed and classified as SEOC papillary, SEOC poorly differentiated papillary, or undifferentiated carcinoma (see Data S1). There was a high concordance in the classification of donor and the P1 and P2 tumors, although a trend towards the loss of well differentiated SEOC histology was observed with increased passage number (Fig. 1B, Table 1). As with the *de novo* SEOC mouse model and human disease, a substantial percentage of mice with orthotopic tumors developed abdominal carcinomatosis (average  = 32%) or distant metastases (average  = 40%).

To monitor passaged tumors at the molecular level, we compared transcriptional profiles of a previously published dataset [26] comprised of normal ovarian surface epithelium and primary SEOCs with a newly generated set of matching primary SEOC and derived orthotopic tumors. By principal component analysis (PCA), normal samples clearly separated from the tumors, while passaged tumors clustered with primary tumors indicating their resemblance to the de novo SEOCs (Fig. 1C). Additionally, cluster analysis of merged human and mouse data using classifier gene sets showed that passaged tumors, similarly to primary tumors, represented all 4 subgroups of human SEOC originally identified in the TCGA study [5] (Fig. 1D). Therefore, the orthotopic transplantable ovarian cancer models recapitulate the human disease on molecular and histopathological levels.

### Murine ovarian carcinoma cells display a drug sensitivity profile similar to human cell lines

For comparison of drug treatment response in murine and human cells, we measured cytotoxicity following exposure to cisplatin and olaparib in established human ovarian carcinoma cells and tumor cell cultures derived from mouse primary SEOCs and ascites (Fig. 1A). While the human line UWB1.289 expresses a truncated inactive BRCA1 protein, the BRCA1 status in human lines 1A9 (a subclone of line A2780), 1A9CP80 and HeyA8 is unknown. To determine if these human cell lines were capable of RAD51 recruitment and therefore homologous recombination-mediated repair, we assessed radiation-induced foci formation of γH2AX and RAD51. While γH2AX marks sites of DSBs independent of BRCA1 functionality, RAD51 recruitment to DSBs is BRCA1-dependent. 1A9CP80 and HeyA8 cells subjected to 10Gy irradiation and stained 6 hours later, showed profound formation of RAD51 and γH2AX foci. In contrast, complete absence of RAD51 foci was seen in 1A9 and UWB1.289 cells (Fig. S1), indicating the lack of BRCA1 functionality.

**Figure 2:**
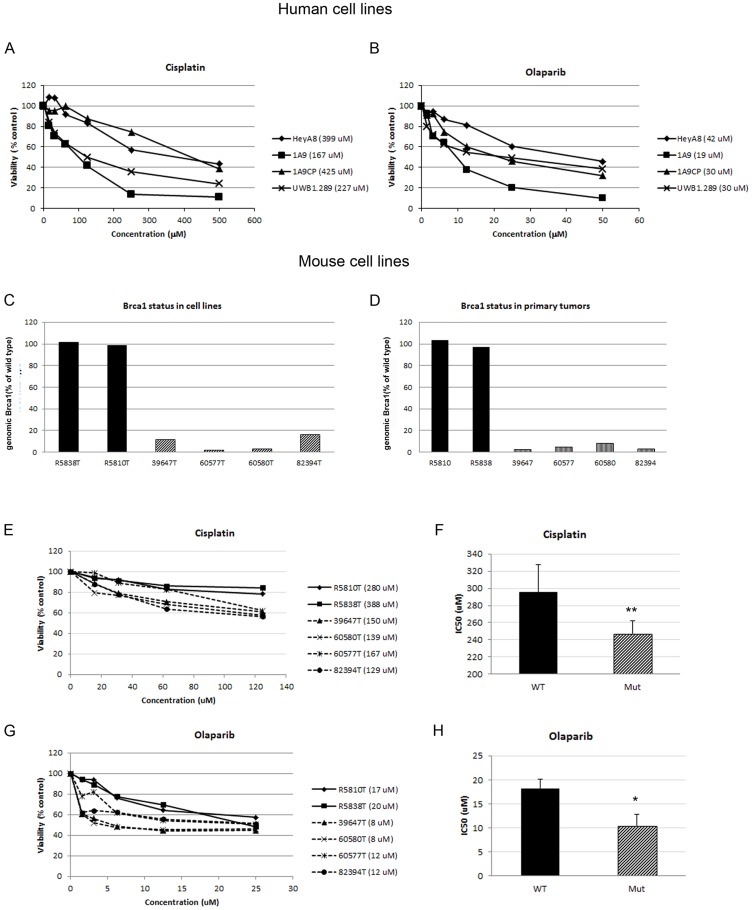
*In vitro* sensitivity of human and mouse ovarian epithelial carcinoma cells to anti-cancer treatment. Human (A, B) and murine (C–H) ovarian cancer cells were exposed to cisplatin, olaparib or vehicle for 7 days after which cell viability was measured using XTT reagent. Proportional viability was calculated by comparing the drugs with vehicle controls whose viability was assumed to be 100%. C. Panel of murine cell lines was selected based on *Brca1* status containing *Brca1*-deficient (striped bar) and –wild type lines (filled bars) maintaining similar *Brca1* expression as their tumors of origin (D). IC50 values for individual cancer cell lines are shown in the legends (A, B, E, G, parenthesis behind the cell line designation). Comparison of the IC50 for cisplatin (F) and olaparib (H) in wild type (*TgK18G_T121_^tg/+^/p53^Δ/Δ^*; R5810T, R5838T) and mutant (*TgK18G_T121_^tg/+^/Brca1^Δ/Δ^/p53^Δ/Δ^*; 39647T, 60577T, 60580T, 82394T) murine cell lines shows significant difference between the genotypes (T-test, 333.7±54.1 vs 146.0±8.2, p<0.01 and 18.2±1.4 vs 10.4±1.2, p<0.05, respectively for drugs). The average and standard error is shown.

As previously reported [27]–[29] UWB1.289 and 1A9 were most sensitive to treatment with cisplatin, while the IC50s of lines 1A9CP80 and HeyA8 were generally 2–2.5 times greater than their sensitive counterparts (Fig. 2A). Olaparib treatment produced the greatest cytotoxicity in 1A9 cells, while 1A9CP80, HeyA8 and UWB1.289 were similar and about 2-fold less sensitive (Fig. 2B).

For comparative cytotoxicity assays, mouse primary SEOC cells lines, harboring either wild-type *Brca1* or the deletion mutant, were cultured. As reported previously [31], *BRCA1*-deficient cells grow poorly in culture. Thus, because Cre-mediated recombination in vivo is not 100%, there is a strong *in vitro* selection for cells with insufficient *Brca1* deletion in cell lines derived from predominantly *Brca1* deficient tumors [31]. Out of 32 cell lines screened, four cell lines that maintained the lowest *Brca1* expression levels (below 20%) relative to the *Brca1* wild type lines were chosen to represent *Brca1*-deficiency (Fig. 2C). These levels of *Brca1* were comparable to their parental tumors (Fig. 2 D). *Brca1* deficiency was also confirmed by functional assay where irradiated cells were unable to recruit RAD51 to the sites of DSBs (Fig. S2). *Brca1*-deficient cell lines (39647, 60580, 60577 and 82394; all *TgK18G_T121_^tg/+^/p53^Δ/Δ^/Brca1^Δ/Δ^* genotype) were approximately 2 times more sensitive to cisplatin than *Brca1*-wild type lines (R5810 and R5838; both *TgK18G_T121_^tg/+^/p53^Δ/Δ^* genotype; Fig. 2E–F). Similarly, treatment with olaparib showed greater potency in *Brca1*-deficient than *Brca1*-wild type cell lines (Fig. 2 G–H). Our results indicate that these cell lines replicate relative sensitivities of human tumors where *BRCA* mutations are found more frequently in platinum-sensitive than platinum-resistant disease [32], [33].

### Orthotopic transplant models recapitulate *Brca*-related differences in treatment response observed in patients

**Figure 3:**
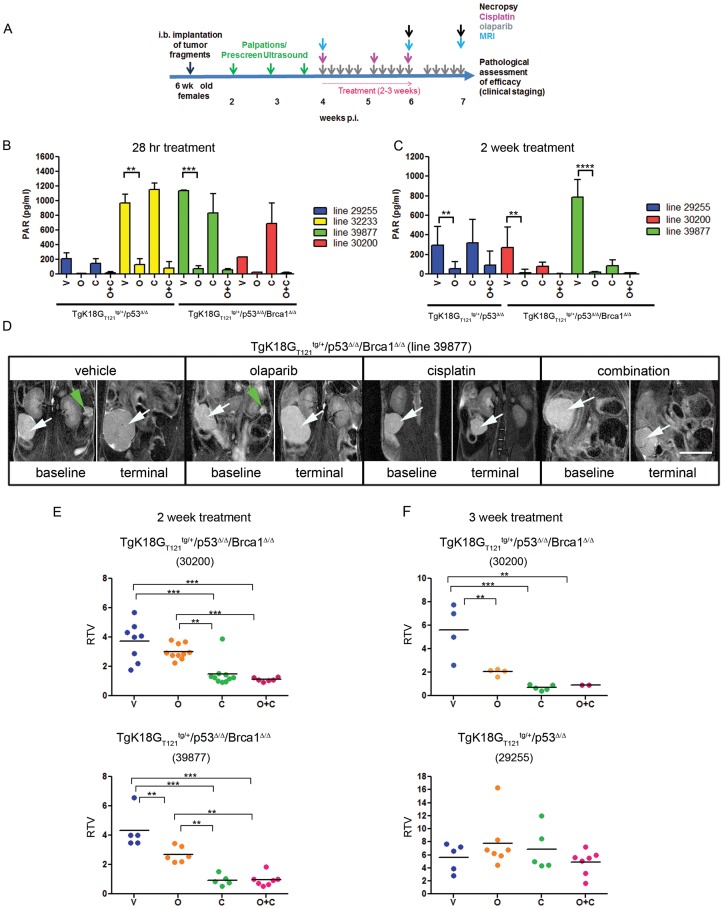
Quantification of tumor progression in orthotopic SEOC models and treatment with cisplatin and/or olaparib. A, Schematics of dosing regimen and imaging in efficacy studies. B, Inhibition of PAR formation in tumor lysates treated with olaparib for 2(C). The average and standard error is shown. D, Representative MR images before and after 2 weeks of treatment with vehicle, olaparib, cisplatin or combination of olaparib and cisplatin are shown. Scale bar represents 1 cm. White arrows point to the tumors, green arrows point to contralateral ovaries. MRI based quantification of tumor volume changes expressed as RTV following 2 week (E) and 3 week (F) treatment regimen. Statistical differences between groups were analyzed by one-way ANOVA and Tukey's multiple comparisons test. Each point represents one animal. V; vehicle, O; olaparib, C; cisplatin, O+C; olaparib and cisplatin.

Ideally, for effective use of any preclinical model in development of human therapeutics and treatment-associated biomarkers, predictability of known human responses should be established. To this end, we evaluated responses in the *Brca1*-deficient and -wild type SEOC models to cisplatin and olaparib. First, to determine an optimal dosing schedule, we examined tumor growth kinetics via serial magnetic resonance (MR) imaging (Fig. S3). Recipient animals developed small ovarian tumors of 70 to 80 mm^3^ (∼0.5 cm in diameter) by 21 to 28 days post implantation (p. i.) followed by rapid tumor growth. The animals became moribund due to large ovarian tumors and ascites by 40 to 60 days p.i. For efficacy studies, treatment was initiated once a small (>80 mm^3^) tumor mass had developed in order to mimic patients with residual tumor burden following de-bulking surgery (study design depicted in Fig. 3A).

To determine in vivo activity and target modulation by olaparib in the orthotopic model, we assayed for PAR levels in treated tumors. Tumor lysates from all animals receiving olaparib treatment had greatly reduced PAR levels after short-term (28 hrs; p<0.01 for line 32233, p<0.001 for line 30200) and long-term (2 weeks; p<0.01 for line 29255 and 30200, p<0.0001 for line 39877) dosing compared to mice treated with vehicle or cisplatin (Fig. 3B, C), indicating that tumor exposure to olaparib was sufficient for PARP inhibition. As expected, there was no difference in PAR levels between *Brca1*-deficient and wild type tumors, consistent with PAR formation occurring upstream of BRCA1, independent of the state of the HR repair mechanism.

We assessed the efficacy of olaparib, cisplatin and their combination in the *Brca1*-deficient models after two weeks of treatment (Fig. 3D, E) by measuring relative tumor volumes (RTV), the tumor volume changes from baseline (pre-dosing) to the end-point as determined by MRI. Each point in graph represents an individual mouse. All 13 vehicle-treated animals from 2 independent tumor models showed rapid tumor growth within 2 weeks, with 3 animals being sacrificed before day 14 due to deteriorating health associated with tumor growth (Fig. 3D, E). Treatment with olaparib resulted in modest reduction of tumor growth in the *Brca1*-deficient 30200 tumor model and in significant reduction in model 39877 (p<0.01). However, in both cases the reduction in RTV was more profound when treated with cisplatin (p<0.001) and the combination of cisplatin and olaparib compared to olaparib alone (p<0.01) (Fig. 3D, E).

To investigate whether response to treatment was dependent on *Brca1* status, we performed a 3-week treatment study on the *Brca1*-deficient model 30200 and a *Brca1*-wild type model 29255. Six of 9 vehicle-treated mice from both lines became moribund before day 21. Cisplatin mono- or combination therapy was equally effective at the 3 and 2 week time points. However, extended treatment of *Brca1*-deficient tumors with olaparib resulted in greater tumor reduction compared to vehicle treated tumors (2.037±0.282 RTV vs 5.598±2.296 RTV, p<0.01) at 3 compared to 2-week treatment (3.008±0.509 vs 3.710±1.325 RTV, ns), suggesting that a threshold of DNA damage is required for optimal potency. In contrast, the *Brca1*-wild type tumor model did not respond to olaparib despite PARP inhibition in tumors, indicating the importance of damaged HR repair to olaparib efficacy (Fig. 3B, F). Cisplatin therapy was equally ineffective in *Brca1-*wild type tumors (Fig. 3F). *Brca1* genotype-specific responses to platinum drugs as well as olaparib have also been documented in patients [34]–[37], therefore the orthotopic model is a useful tool for examining these differential responses and predicting potential outcomes.

### Histopathological changes resulting from olaparib and cisplatin treatment reflect differences observed in tumor volumes

**Figure 4:**
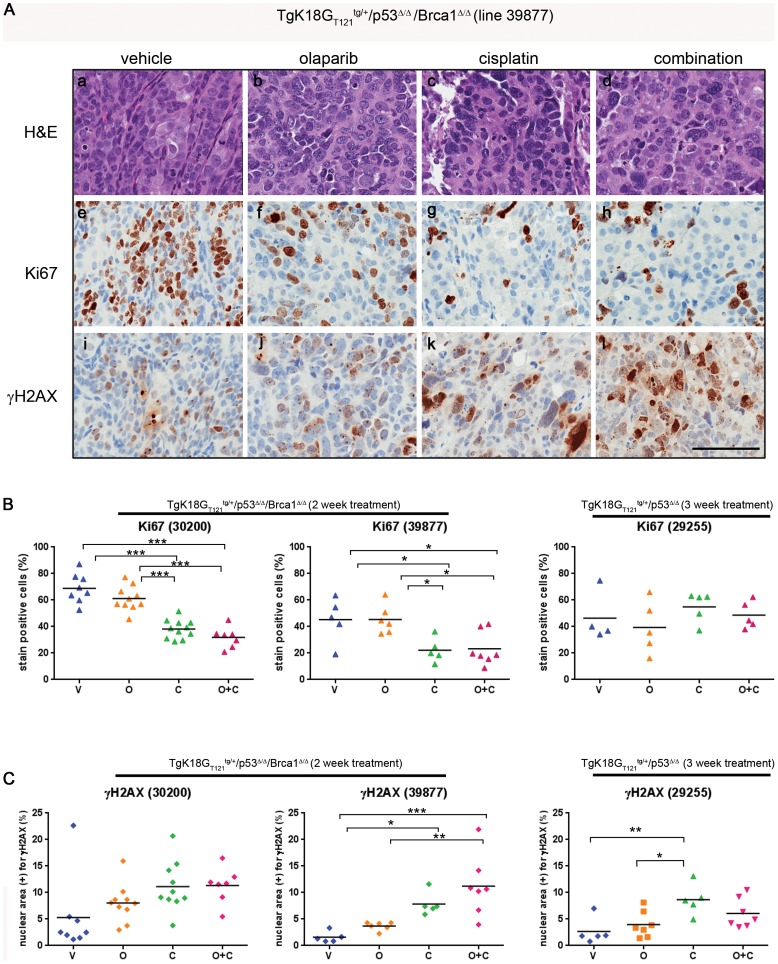
Assessment of histopathological changes induced by drug treatment in *Brca1*-wild type and -deficient tumor lines. A, An example of histology and IHC of *TgK18G_T121_^tg/+^/Brca1^Δ/Δ^/p53^Δ/Δ^* tumors treated with cisplatin and/or olaparib (a–l). Tumor line 39877 was sensitive to drug treatment, which resulted in decreased proliferation (Ki67) (A e–h) and increased DNA damage (γ-H2AX) (A i–l). Olaparib treatment resulted in increased nuclear size and pleiomorphism (A, b, f, j) as well as decreased degree of papillary differentiation. Note the marked increase in tumor multinucleated giant cells in the cisplatin (A, c, g, k) and combination treated (A, d, h, l). Scale bar represents 100 µm. Quantitative analysis of Ki67 (B), and γH2AX (C) in 3 different tumor lines. Proliferation rate is expressed as the percentage of Ki67 positive nuclei (brown-DAB) to the total number of nuclei in the tumor section (brown-DAB + blue-hematoxylin counter-stained negative nuclei). γH2AX is expressed as the percentage of total nuclear area (blue-hematoxylin counter-stain) to the total area positive for γH2AX (brown-DAB). Statistical differences between groups were analyzed by one-way ANOVA and Tukey's multiple comparisons test. Each point represents one animal. V; vehicle, O; olaparib, C; cisplatin, O+C; olaparib and cisplatin.

To assess the effect of drug treatment at the cellular level, H&E-stained tumor sections were analyzed for histological changes (Fig. 4Aa–l). In both *Brca1*-deficient models, treatment with olaparib resulted in less differentiated SEOC with increased nuclear size and pleomorphism compared to the well to moderately differentiated papillary structures of the ovarian tumors in vehicle-treated mice (Fig. 4A a, b). In contrast, cisplatin monotherapy had a more profound effect on tumor histology (Fig. 4Ac), producing poorly differentiated papillary SEOC or undifferentiated carcinoma with an increase in tumor stroma, necrotic foci, and apoptotic cells with a high degree of nuclear pleiomorphism and the presence of atypical multinucleated tumor giant cells. Treatment of the *Brca1*-deficient models with combination of cisplatin and olaparib resulted in histology very similar to that of cisplatin alone-treated tumors (Fig. 4Ad). In contrast, minimal drug treatment effects were observed in the *Brca1*-wild type tumor model indicating an overall concordance of histopathological and tumor volume changes following drug treatment (Fig. S4).

Proliferation rates and DNA damage levels in tumors from treated animals were assessed by IHC. There was a general decrease in proliferation in tumors treated with cisplatin or the combination of cisplatin and olaparib compared to vehicle in both *Brca1*-deficient tumor models, but not in the *Brca1*-wild type model (Fig. 4B), even though *Brca1*-wild type model was treated for a longer time (3 weeks). Consistent with the lack of tumor volume changes, treatment with olaparib did not significantly decrease proliferation in any of the models. DNA damage as assayed by γH2AX staining generally increased in all tumors after cisplatin or combination treatment (Fig. 4C).

In general, decrease in cell proliferation and increase in DNA damage correlated with tumor growth inhibition observed in *Brca1*-deficient lines. In contrast, increased DNA damage after cisplatin treatment in *Brca1-*wild type tumors did not result in decreased tumor volume likely due to effective DNA repair.

### Long term olaparib and/or cisplatin treatment provides progression-free benefit but results in relapse of animals harboring *Brca1*-deficient tumors

In the clinic, long term treatment with olaparib resulted in significant improvement in progression-free survival among patients with SEOC [16]. Given successful tumor growth inhibition by cisplatin and the cisplatin/olaparib combination in *Brca1*-deficient orthotopic models, we asked whether responses to long term treatment with olaparib would reflect these human outcomes. We examined the impact of mono- or combinatorial long-term therapy on survival in a cohort that received *Brca1*-deficient orthotopic ovarian tumor transplants. Tumor-bearing animals from model 39877 were continuously treated with each of the 4 dosing regimens for 10 weeks. To monitor tumor volumes, the animals were imaged biweekly using ultrasound for up to 16 weeks. A threshold image segmentation technique was used to ensure comparability of ovarian volumes deduced from MR and US images. RTV changes determined by ultrasound (US) following 2 weeks of dosing were similar to the previous experiment wherein RTV was assessed by MR image analysis (Fig. S5).

**Figure 5:**
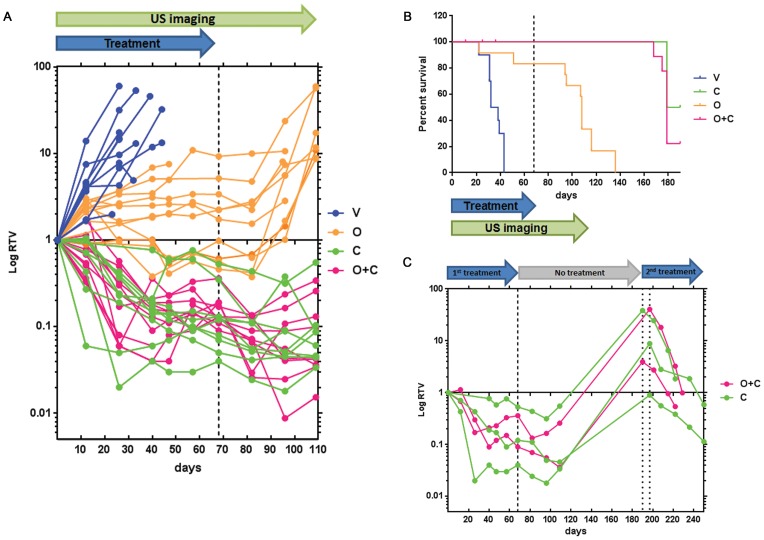
Effect of long term treatment on mouse survival. A, RTV measurements in survival study comparing the effect of different treatments on tumor development. RTV compares tumor volume at any given time point to the baseline (pre-dosing) tumor volume. Each point represents an individual animal. B, Kaplan-Mayer graph of mouse survival after long term treatment. Dashed line represents cessation of treatment at Day 68. Three out of 12 mice treated with combination therapy died early in the study and although they presented with small ovarian tumors they did not succumb to metastatic SEOC. Histopathological signs of mild nephrosis as a result of cytotoxicity have been observed in their tissues [V; vehicle (n = 10), O; olaparib (n = 12), C; cisplatin (n = 10), O+C; olaparib and cisplatin (n = 12)]. C, effect of second round of platinum or combination treatment following the tumor relapses in mice. Tumor volumes were determined by US imaging. Dashed line represents end of first round of treatment, dotted lines represent start of the second round of treatment (two different start dates for 2 different mouse groups).

All 10 vehicle-treated animals were euthanized by day 44 due to metastatic SEOC (Fig. 5 A, B). Treatment with olaparib resulted in significant reduction of tumor growth, compared to vehicle (2.25±0.06 RTV, n = 12 vs 4.93±3.55 RTV, n = 10, p<0.01) as soon as 12 days after start of the treatment. This suppression persisted throughout treatment, indicating a clear progression free survival benefit provided by olaparib. However, all mice eventually succumbed to SEOC within 68 days after treatment cessation. Treatment with cisplatin (0.82±0.6 RTV, n = 10) or cisplatin in combination with olaparib (0.95±0.65 RTV, n = 11) resulted in substantial reduction of tumor growth compared to vehicle-treated animals (4.93±3.55 RTV, n = 10, p<0.001), 12 days post treatment start, with complete regression of some tumors at later time points. There was some toxicity evident in the combination-treated mice, as 3 mice died in that group after 11–36 days of treatment. Mice treated with olaparib survived significantly longer (ANOVA p<0.001; median survival 108 days) than vehicle-treated mice (median survival 35 days), but not as long as cisplatin (median survival 184.5 days) or combination-treated mice (median survival 179 days). In the latter two groups, no re-growth of tumors was evident by US imaging 40 days after cessation of treatment (treatment ended at Day 68). However, 17 out of 22 mice succumbed to SEOC within 111 days after the last treatment (Fig. 5B), indicating that residual tumor, not detectable by US imaging, remained. Mice that survived were enrolled into a second treatment cycle with cisplatin (n = 3) or cisplatin/olaparib (n = 2) following tumor re-growth. Treatment was carried out for up to 8 weeks and tumor growth was monitored by biweekly US imaging. The second treatment cycle again resulted in tumor regression, signifying that tumors were still sensitive to platinum treatment (Fig. 5C) and thus might represent a fraction of patients that respond to second- or third-line of platinum therapy [38].

As with short term treatment, prolonged treatment of orthotopic *Brca1*-wild type ovarian tumors with cisplatin, olaparib or their combination did not result in tumor regression or tumor growth arrest (Fig. S6 A, B).

These results indicate that olaparib therapy can be used in combination as a maintenance therapy suppressing tumor growth in *Brca1*-deficient but not *Brca1*-wild type tumors. Given the increase in survival seen with olaparib treatment alone, the orthotopic model can be used to assess alternate dosing regimens designed to avoid toxicity associated with cisplatin treatment.

## Discussion

The development and characterization of mouse ovarian orthotopic tumor transplant models resembling human SEOC offers a significant improvement over currently available options for preclinical testing of new therapeutics for treatment of ovarian cancer. Compared to our previously generated *de novo* GEM models for SEOC, these orthotopic transplant models exhibit accelerated disease onset and can be efficiently produced as synchronized cohorts of tumor-bearing animals with intact immune systems for relevant side-by-side comparison of therapeutic regimens and biomarker development in *Brca1*-wild type and –deficient tumors.

Murine cell lines derived from primary ovarian tumors and human ovarian cancer cell lines responded similarly to cisplatin and olaparib treatment. *Brca1*-deficient murine cell lines displayed increased sensitivity to both compounds as was observed in the human *BRCA1*-deficient line UWB1.289. The human line 1A9 was more sensitive to cisplatin as well, and although its *BRCA1* status is unknown, we show by a functional assay that homologous recombination repair mechanism is impaired (Fig. S1). Overall, the mouse tumor cell lines exhibit comparable sensitivities to human SEOC cells when treated with known chemotherapeutic agents and represent useful tools for *in vitro* screening the potency of novel therapeutic drugs. Compared to human cell lines in which the BRCA status is often unknown, murine cell lines with defined genotypes provide the opportunity to detect genotype-specific differences in therapeutic responses.

In the syngeneic orthotopic transplant models reported herein, *Brca1* deficiency rendered tumors more sensitive to platinum treatment than *Brca-1* wild type counterparts. These findings are analogous to clinical results with hereditary *BRCA*-mutant cancers that also have increased sensitivity to platinum chemotherapy [34]–[36], [39], [40], thus underscoring the potential importance of patient genotype analysis even prior to non-targeted therapies. Reduced sensitivity of mouse *Brca1-*wild type tumors to platinum treatment cannot be attributed to slower tumor growth, since growth kinetics are similar between *Brca1*-deficient and -wild type tumors subsequent to establishment and prior to treatment (Fig. S3). Short term treatments (2–3 weeks) of *Brca1*-deficient tumors resulted in significant reduction of tumor volumes compared to vehicle treated mice, corresponding to the observed decreased proliferation and increased DNA damage. Moreover, prolonged 10-week treatment often resulted in complete regression of detectable tumors. Ultimate tumor regrowth suggests that a remnant of cancerous cells survived the long-term treatment. Similarly to previous reports [41], [42], these recurrent tumors responded to a second round of platinum treatment, suggesting the presence of tumor-initiating cells that are initially less vulnerable to cisplatin effects due to the cell cycle arrest or, possibly, presence of cisplatin resistant cells that produce tumors with increased sensitivity upon differentiation. These models thus represent a valuable resource for testing treatments targeting residual cells after platinum therapy.

The continued sensitivity to cisplatin that we and others [42] observed in mouse models differs from outcomes seen in ovarian cancer patients, where there is considerable heterogeneity observed in response to second-line platinum-based chemotherapy, and other therapeutic regimens have to be considered for recurrent platinum-resistant or refractory disease [38],[43],[44]. These differences may be due to the discrepancy in *BRCA1/2* deficiency between GEMs and human patients. While BRCA1/2 deficiency in humans is most frequently a result of mutations, promoter hypermethylation or other unknown mechanisms, in GEMs it is a result of engineered large deletions in *Brca1/2* genes. Secondary mutations in the *BRCA1* gene that restored open reading frame of mutated *Brca1* allele have been observed in ovarian tumors with resistance to platinum [43]. This suggests that down regulation of *BRCA1/2* genes causes initial sensitivity to cisplatin and restoration of functional BRCA1/2 expression, either by secondary mutations, promoter demethylation, or other mechanisms, leads to acquired resistance. Generally in GEMs this restoration of BRCA function through secondary mutations in *BRCA* gene is not possible due to large deletions in the gene [42]. However, other mechanisms for acquired resistance, mutations or epigenetic alternations in genes other than *Brca1/2*, has been shown to increase resistance to DNA-damaging agents in GEMs [45], [46]. GEM models that develop resistance to particular therapeutics thus become an especially useful tool to study the mechanisms of the acquired resistance [45]. Multiple treatment cycles in the orthotopic models reported here are underway and may also result in increased resistance that can be exploited to develop alternative therapeutic regimens.

Although short-term treatment of murine orthotopic tumors with olaparib did not provide any significant benefit, long-term treatment significantly suppressed tumor development in *Brca1-* deficient but not in *Brca1*-wild type tumors, similar to the progression free survival benefit observed in patients [15], [16], [47]. However, complete tumor regression was not observed in this group. Moreover, tumors readily relapsed after cessation of treatment suggesting that olaparib therapy, similar to trials in patients, did not translate into an overall survival benefit [16]. Recently, a retrospective analysis of olaparib Phase II trial data confirmed that olaparib would be most beneficial for patients with deleterious *BRCA1/2* mutations [37]. Astra Zeneca recently announced the progression of olaparib into Phase III studies (SOLO1 and SOLO2) that will assess olaparib's efficacy as a maintenance therapy for patients who have previously received platinum-based chemotherapy. Crucially, the trial inclusion criteria include the requirement that patient tumors carry deleterious or suspected deleterious *BRCA1/2* mutations.

In contrast to GEMMs for breast cancer [46], combined treatment with cisplatin and olaparib in the SEOC allograft models did not result in increased efficacy over cisplatin alone. This difference could be attributed to differences in dosing regimens between the two studies. While mice with mammary cancers were given repeated cycles of single dose of cisplatin after each relapse, our study treated allografts continuously, which resulted in very efficient tumor regression. Thus, any added benefit of olaparib may have been masked by the efficiency of cisplatin. We also found the potential for adverse effects in the combination treatment. Given that olaparib treatment alone increased survival in the orthotopic model, future studies in the Brca1-deficient model should compare alternate dosing regimens such as initial dosing with cisplatin, followed by olaparib single treatment, to maximize potential efficacy while minimizing toxicity.

In summary, we have established tractable preclinical ovarian cancer models suitable for efficacy testing of existing and investigative therapeutics tailored to the genetic status of tumors. The ability to rapidly assess efficacy in these models provides an asset to future ovarian cancer drug discovery efforts. Novel targeted small molecule drugs may be evaluated in combination with standard of care, as in this study. Additionally, the presence of tumor cells refractory to initial cisplatin treatment allows for the possibility of studying drug resistance. Finally, the use of immunocompetent, syngeneic transplant models allows for the evaluation of drugs that may affect the tumor microenvironment, including the impact of an intact immune system for both immunomodulatory and signaling targeted therapies.

## Supporting Information

Figure S1 Functional assay for status of homologous recombination in human ovarian carcinoma cell lines. Non-irradiated and cells treated with a single dose of 10Gy were stained for phosphohistone H2A.X (to visualize sites of DSBs) and RAD51 (to visualize foci of BRCA1-dependent recruitment of RAD51 to DSBs). RAD51-containing foci are not present in cells that exhibit increased sensitivity to cisplatin (1A9 and UWB1.289). White arrows are pointing to cells with RAD51 foci.
(TIF)

Figure S2 Functional assay for status of homologous recombination in murine ovarian carcinoma cell lines. Non-irradiated and cells treated with a single dose of 10Gy were stained for phosphohistone H2A.X (to visualize sites of DSBs) and RAD51 (to visualize foci of BRCA1-dependent recruitment of RAD51 to DSBs). RAD51-containing foci are not present in Brca1-deficient cells that exhibit increased sensitivity to cisplatin (39647, 60577, 82394, 60580). White arrows are pointing to cells with RAD51 foci.
(TIF)

Figure S3 Kinetics of the orthotopic tumor growth. To determine the optimal time point for beginning of treatments in efficacy studies, the tumor growth kinetics was first assessed for 2 K18G_T121_^tg/+^/p53^Δ/Δ^/Brca1^Δ/Δ^ tumor lines (A, B) by tumor volume measurements over the period of time. The optimal starting tumor volume was determined to be 0.07–0.2 cm3, which would allow for at let 2 weeks of treatment before the mice had to be euthanized due to tumor burden and/or presence of ascites. C, Kinetics of the tumor development in K18G_T121_^tg/+^/p53^Δ/Δ^/Brca1^Δ/Δ^ and K18G_T121_^tg/+^/p53^Δ/Δ^ tumor lines indicates delayed start for tumors wild type for Brca1 but very similar growth rates once the tumors are established.
(TIF)

Figure S4 Assessment of histopathological changes induced by drug treatment in Brca1-wild type tumor line. An example of histology and IHC of Brca1-wild type tumors treated with cisplatin and/or olaparib (a–l). Tumors did not respond to treatment with olaparib and/or cisplatin with marked decrease in cell proliferation (e–h). Increased DNA damage observed after cisplatin treatment did not result in decreased tumor volume likely due to intact DNA repair mechanism.
(TIF)

Figure S5 Progression of the orthotopic tumor development in tumor line 39877 determined from ultrasound imaging. Tumor volume measurements were obtained from ultrasound image sequences before beginning of treatment (0) and after 2 weeks of treatment (2). RTV compares tumor volume at any given time point to the baseline (0) tumor volume. The results are comparable with those obtained in efficacy study where tumor volumes were determined from MR images (Fig. 3).
(TIF)

Figure S6 Effect of long-term treatment on Brca1-wild type tumors. Prolonged treatment with cisplatin, olaparib or their combination had minimal effect on tumor growth (A) or survival (B) of mice implanetd with Brca1-wild type tumor (line 29255). O; olaparib (n = 5), C; cisplatin (n = 5), O+C; olaparib and cisplatin (n = 3), V; vehicle (n = 4).
(TIF)

Table S1 Summary of the results obtained from orthotopic grafting of cultured cell lines.
(DOCX)

Data S1 Supplementary Data contain material and methods for establishment of murine primary ovarian cancer cell lines, methods and results for cell implantations, methods for imaging and tumor volume measurements, tissue collection, pathological and IHC analysis, quantitative analysis of IHC stains, immunofluorescent staining of human and murine cells and quantitative PCR analysis of *Brca1* status.
(DOCX)

## References

[1] American Cancer Society (2012). Cancer facts & figures, 2012. Atlanta.

[2] McGuire WP 3rd, Markman M (2003) Primary ovarian cancer chemotherapy: current standards of care. Br J Cancer 89 Suppl 3S3–8. 10.1038/sj.bjc.6601494 PMC2750616 14661040

[3] Quinn JE, Carser JE, James CR, Kennedy RD, Harkin DP (2009) BRCA1 and implications for response to chemotherapy in ovarian cancer. Gynecol Oncol 113: 134–142. 19168207 10.1016/j.ygyno.2008.12.015

[4] Yap TA, Carden CP, Kaye SB (2009) Beyond chemotherapy: targeted therapies in ovarian cancer. Nat Rev Cancer 9: 167–181. 19238149 10.1038/nrc2583

[5] Cancer Genome Atlas Research Network (2011) Integrated genomic analyses of ovarian carcinoma. Nature 474: 609–615. 21720365 10.1038/nature10166 PMC3163504

[6] Roy R, Chun J, Powell SN (2012) BRCA1 and BRCA2: different roles in a common pathway of genome protection. Nat Rev Cancer 12: 68–78. 10.1038/nrc3181 PMC4972490 22193408

[7] Dedes KJ, Wilkerson PM, Wetterskog D, Weigelt B, Ashworth A, et al (2011) Synthetic lethality of PARP inhibition in cancers lacking BRCA1 and BRCA2 mutations. Cell Cycle 10: 1192–1199. 21487248 10.4161/cc.10.8.15273 PMC3117132

[8] Chernikova SB, Game JC, Brown JM (2012) Inhibiting homologous recombination for cancer therapy. Cancer Biol Ther 13: 61–68. 22336907 10.4161/cbt.13.2.18872 PMC3336066

[9] Schreiber V, Dantzer F, Ame JC, de Murcia G (2006) Poly(ADP-ribose): novel functions for an old molecule. Nat Rev Mol Cell Biol 7: 517–528. 16829982 10.1038/nrm1963

[10] Lord CJ, Ashworth A (2008) Targeted therapy for cancer using PARP inhibitors. Curr Opin Pharmacol 8: 363–369. 18644251 10.1016/j.coph.2008.06.016

[11] Plummer ER (2006) Inhibition of poly(ADP-ribose) polymerase in cancer. Curr Opin Pharmacol 6: 364–368. 16753340 10.1016/j.coph.2006.02.004

[12] Menear KA, Adcock C, Boulter R, Cockcroft XL, Copsey L, et al (2008) 4-[3-(4-cyclopropanecarbonylpiperazine-1-carbonyl)-4-fluorobenzyl]-2H-phthalazin- 1-one: a novel bioavailable inhibitor of poly(ADP-ribose) polymerase-1. J Med Chem 51: 6581–6591. 18800822 10.1021/jm8001263

[13] Annunziata CM, O'Shaughnessy J (2010) Poly (ADP-ribose) polymerase as a novel therapeutic target in cancer. Clin Cancer Res 16: 4517–4526. 20823142 10.1158/1078-0432.CCR-10-0526 PMC2981097

[14] Khan OA, Gore M, Lorigan P, Stone J, Greystoke A, et al (2011) A phase I study of the safety and tolerability of olaparib (AZD2281, KU0059436) and dacarbazine in patients with advanced solid tumours. Br J Cancer 104: 750–755. 21326243 10.1038/bjc.2011.8 PMC3048218

[15] Gelmon KA, Tischkowitz M, Mackay H, Swenerton K, Robidoux A, et al (2011) Olaparib in patients with recurrent high-grade serous or poorly differentiated ovarian carcinoma or triple-negative breast cancer: a phase 2, multicentre, open-label, non-randomised study. Lancet Oncol 12: 852–861. 21862407 10.1016/S1470-2045(11)70214-5

[16] Ledermann J, Harter P, Gourley C, Friedlander M, Vergote I, et al (2012) Olaparib maintenance therapy in platinum-sensitive relapsed ovarian cancer. N Engl J Med 366: 1382–1392. 22452356 10.1056/NEJMoa1105535

[17] Hay T, Matthews JR, Pietzka L, Lau A, Cranston A, et al (2009) Poly(ADP-ribose) polymerase-1 inhibitor treatment regresses autochthonous Brca2/p53-mutant mammary tumors in vivo and delays tumor relapse in combination with carboplatin. Cancer Res 69: 3850–3855. 19383921 10.1158/0008-5472.CAN-08-2388

[18] Fong PC, Yap TA, Boss DS, Carden CP, Mergui-Roelvink M, et al (2010) Poly(ADP)-ribose polymerase inhibition: frequent durable responses in BRCA carrier ovarian cancer correlating with platinum-free interval. J Clin Oncol 28: 2512–2519. 20406929 10.1200/JCO.2009.26.9589

[19] Fong PC, Boss DS, Yap TA, Tutt A, Wu P, et al (2009) Inhibition of poly(ADP-ribose) polymerase in tumors from BRCA mutation carriers. N Engl J Med 361: 123–134. 19553641 10.1056/NEJMoa0900212

[20] Kim WY, Sharpless NE (2012) Drug efficacy testing in mice. Curr Top Microbiol Immunol 355: 19–38. 21823029 10.1007/82_2011_160 PMC3732649

[21] Xing D, Orsulic S (2006) A mouse model for the molecular characterization of brca1-associated ovarian carcinoma. Cancer Res 66: 8949–8953. 16982732 10.1158/0008-5472.CAN-06-1495 PMC1802660

[22] Quinn BA, Xiao F, Bickel L, Martin L, Hua X, et al (2010) Development of a syngeneic mouse model of epithelial ovarian cancer. J Ovarian Res 3: 1–24. 20958993 10.1186/1757-2215-3-24 PMC2974672

[23] Connolly DC, Bao R, Nikitin AY, Stephens KC, Poole TW, et al (2003) Female mice chimeric for expression of the simian virus 40 TAg under control of the MISIIR promoter develop epithelial ovarian cancer. Cancer Res 63: 1389–1397. 12649204

[24] Flesken-Nikitin A, Choi KC, Eng JP, Shmidt EN, Nikitin AY (2003) Induction of carcinogenesis by concurrent inactivation of p53 and Rb1 in the mouse ovarian surface epithelium. Cancer Res 63: 3459–3463. 12839925

[25] Orsulic S, Li Y, Soslow RA, Vitale-Cross LA, Gutkind JS, et al (2002) Induction of ovarian cancer by defined multiple genetic changes in a mouse model system. Cancer Cell 1: 53–62. 12086888 10.1016/s1535-6108(01)00002-2 PMC2267863

[26] Szabova L, Yin C, Bupp S, Guerin TM, Schlomer JJ, Householder DB, Baran ML, Yi M, Song Y, Sun W, McDunn JE, Martin PL, Van Dyke T, Difilippantonio S (2012) Perturbation of Rb, p53 and Brca1 or Brca2 cooperate in inducing metastatic serous epithelial ovarian cancer. Cancer Res 72: 4141–4153. 22617326 10.1158/0008-5472.CAN-11-3834 PMC3421072

[27] Buick RN, Pullano R, Trent JM (1985) Comparative properties of five human ovarian adenocarcinoma cell lines. Cancer Res 45: 3668–3676. 4016745

[28] Helleman J, Burger H, Hamelers IH, Boersma AW, de Kroon AI, et al (2006) Impaired cisplatin influx in an A2780 mutant cell line: evidence for a putative, cis-configuration-specific, platinum influx transporter. Cancer Biol Ther 5: 943–949. 16775422 10.4161/cbt.5.8.2876

[29] Fojo T, Farrell N, Ortuzar W, Tanimura H, Weinstein J, et al (2005) Identification of non-cross-resistant platinum compounds with novel cytotoxicity profiles using the NCI anticancer drug screen and clustered image map visualizations. Crit Rev Oncol Hematol 53: 25–34. 15607933 10.1016/j.critrevonc.2004.09.008

[30] Scudiero DA, Shoemaker RH, Paull KD, Monks A, Tierney S, et al (1988) Evaluation of a soluble tetrazolium/formazan assay for cell growth and drug sensitivity in culture using human and other tumor cell lines. Cancer Res 48: 4827–4833. 3409223

[31] Stordal B, Timms K, Farrelly A, Gallagher D, Busschots S, et al (2013) BRCA1/2 mutation analysis in 41 ovarian cell lines reveals only one functionally deleterious BRCA1 mutation. Mol Oncol 7: 567–579. 23415752 10.1016/j.molonc.2012.12.007 PMC4106023

[32] Gallagher DJ, Konner JA, Bell-McGuinn KM, Bhatia J, Sabbatini P, et al (2011) Survival in epithelial ovarian cancer: a multivariate analysis incorporating BRCA mutation status and platinum sensitivity. Ann Oncol 22: 1127–1132. 21084428 10.1093/annonc/mdq577 PMC6267858

[33] Dann RB, DeLoia JA, Timms KM, Zorn KK, Potter J, et al (2012) BRCA1/2 mutations and expression: response to platinum chemotherapy in patients with advanced stage epithelial ovarian cancer. Gynecol Oncol 125: 677–682. 22406760 10.1016/j.ygyno.2012.03.006

[34] Rubin SC, Benjamin I, Behbakht K, Takahashi H, Morgan MA, et al (1996) Clinical and pathological features of ovarian cancer in women with germ-line mutations of BRCA1. N Engl J Med 335: 1413–1416. 8875917 10.1056/NEJM199611073351901

[35] Rigakos G, Razis E (2012) BRCAness: finding the Achilles heel in ovarian cancer. Oncologist 17: 956–962. 22673632 10.1634/theoncologist.2012-0028 PMC3399652

[36] Cass I, Baldwin RL, Varkey T, Moslehi R, Narod SA, et al (2003) Improved survival in women with BRCA-associated ovarian carcinoma. Cancer 97: 2187–2195. 12712470 10.1002/cncr.11310

[37] Ledermann JA, Harter P, Gourley C, Friedlander M, Vergote I, et al (2013) Olaparib maintenance therapy in patients with platinum-sensitive relapsed serous ovarian cancer (SOC) and a BRCA mutation (BRCAm). J Clin Oncol 31: (suppl; abstr 5505)

[38] Markman M, Rothman R, Hakes T, Reichman B, Hoskins W, et al (1991) Second-line platinum therapy in patients with ovarian cancer previously treated with cisplatin. J Clin Oncol 9: 389–393. 1999708 10.1200/JCO.1991.9.3.389

[39] Carser JE, Quinn JE, Michie CO, O'Brien EJ, McCluggage WG, et al (2011) BRCA1 is both a prognostic and predictive biomarker of response to chemotherapy in sporadic epithelial ovarian cancer. Gynecol Oncol 123: 492–498. 21920589 10.1016/j.ygyno.2011.08.017

[40] Boyd J, Sonoda Y, Federici MG, Bogomolniy F, Rhei E, et al (2000) Clinicopathologic features of BRCA-linked and sporadic ovarian cancer. Jama 283: 2260–2265. 10807385 10.1001/jama.283.17.2260

[41] Borst P, Rottenberg S, Jonkers J (2008) How do real tumors become resistant to cisplatin? Cell Cycle 7: 1353–1359. 18418074 10.4161/cc.7.10.5930

[42] Rottenberg S, Nygren AO, Pajic M, van Leeuwen FW, van der Heijden I, et al (2007) Selective induction of chemotherapy resistance of mammary tumors in a conditional mouse model for hereditary breast cancer. Proc Natl Acad Sci U S A 104: 12117–12122. 17626183 10.1073/pnas.0702955104 PMC1914039

[43] Swisher EM, Sakai W, Karlan BY, Wurz K, Urban N, et al (2008) Secondary BRCA1 mutations in BRCA1-mutated ovarian carcinomas with platinum resistance. Cancer Res 68: 2581–2586. 18413725 10.1158/0008-5472.CAN-08-0088 PMC2674369

[44] Markman M, Bookman MA (2000) Second-line treatment of ovarian cancer. Oncologist 5: 26–35. 10706647 10.1634/theoncologist.5-1-26

[45] Jaspers JE, Kersbergen A, Boon U, Sol W, van Deemter L, et al (2013) Loss of 53BP1 causes PARP inhibitor resistance in Brca1-mutated mouse mammary tumors. Cancer Discov 3: 68–81. 23103855 10.1158/2159-8290.CD-12-0049 PMC7518105

[46] Rottenberg S, Jaspers JE, Kersbergen A, van der Burg E, Nygren AO, et al (2008) High sensitivity of BRCA1-deficient mammary tumors to the PARP inhibitor AZD2281 alone and in combination with platinum drugs. Proc Natl Acad Sci U S A 105: 17079–17084. 18971340 10.1073/pnas.0806092105 PMC2579381

[47] Ratner ES, Sartorelli AC, Lin ZP (2012) Poly (ADP-ribose) polymerase inhibitors: on the horizon of tailored and personalized therapies for epithelial ovarian cancer. Curr Opin Oncol 24: 564–571. 22759740 10.1097/CCO.0b013e3283564230 PMC3799945

