# Supplementary figures and images for: Pathway-Specific Engineered Mouse Allograft Models Functionally Recapitulate Human Serous Epithelial Ovarian Cancer
Source: PLoS One. 2014 Apr 18;9(4):e95649. doi: 10.1371/journal.pone.0095649 (PMC3991711; doi:10.1371/journal.pone.0095649)

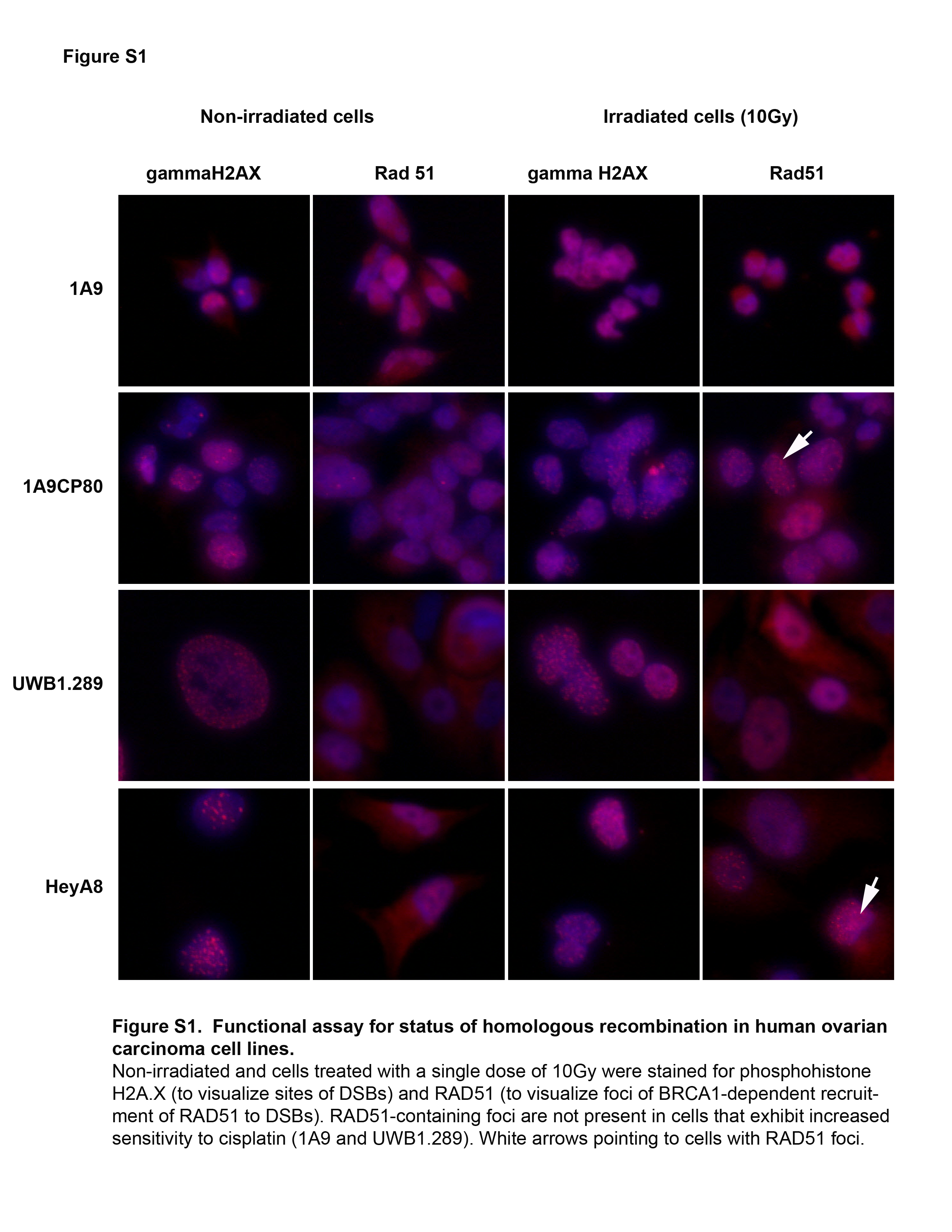

Supplement: Figure S1 — Functional assay for status of homologous recombination in human ovarian carcinoma cell lines. Non-irradiated and cells treated with a single dose of 10Gy were stained for phosphohistone H2A.X (to visualize sites of DSBs) and RAD51 (to visualize foci of BRCA1-dependent recruitment of RAD51 to DSBs). RAD51-containing foci are not present in cells that exhibit increased sensitivity to cisplatin (1A9 and UWB1.289). White arrows are pointing to cells with RAD51 foci. (TIF) [file pone.0095649.s001.tif]

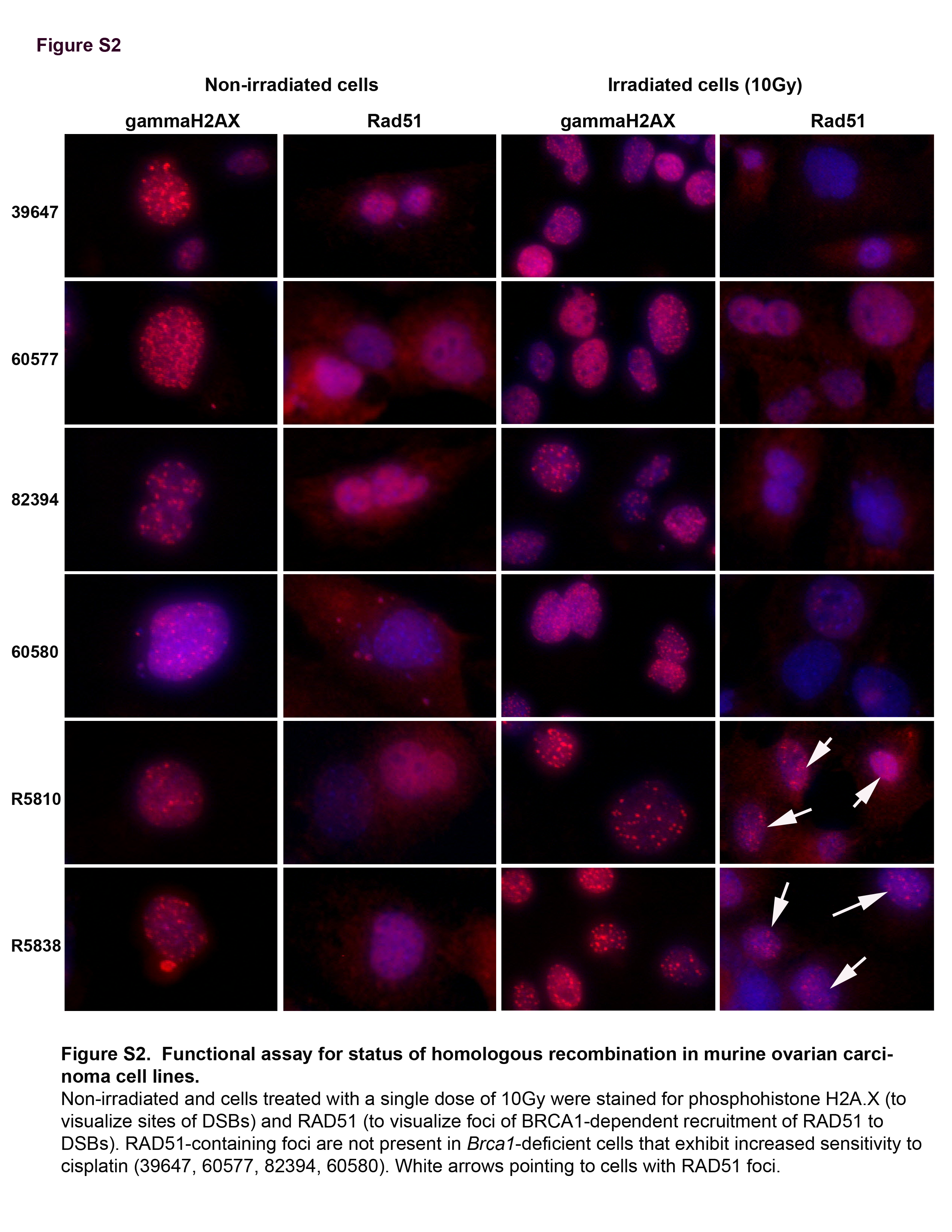

Supplement: Figure S2 — Functional assay for status of homologous recombination in murine ovarian carcinoma cell lines. Non-irradiated and cells treated with a single dose of 10Gy were stained for phosphohistone H2A.X (to visualize sites of DSBs) and RAD51 (to visualize foci of BRCA1-dependent recruitment of RAD51 to DSBs). RAD51-containing foci are not present in Brca1-deficient cells that exhibit increased sensitivity to cisplatin (39647, 60577, 82394, 60580). White arrows are pointing to cells with RAD51 foci. (TIF) [file pone.0095649.s002.tif]

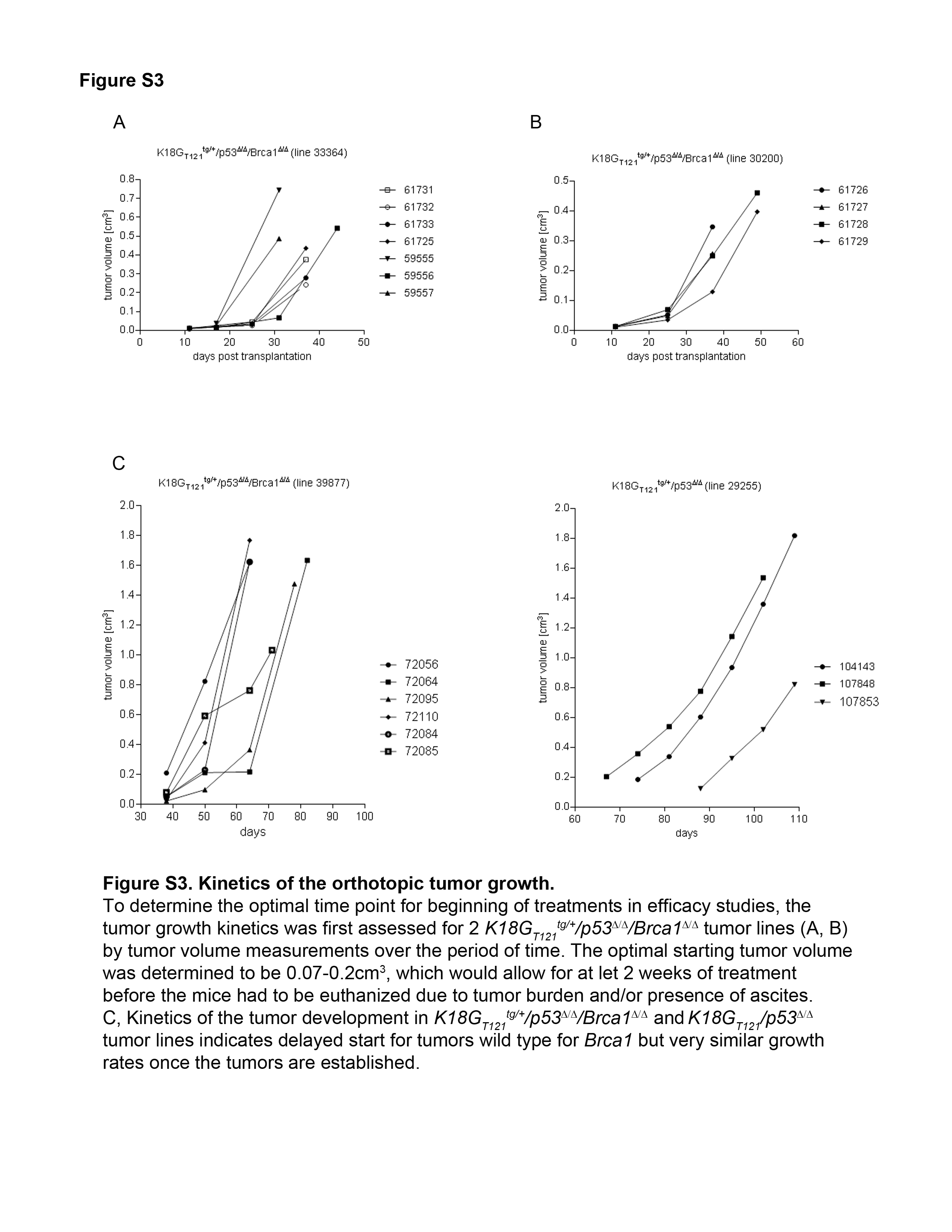

Supplement: Figure S3 — Kinetics of the orthotopic tumor growth. To determine the optimal time point for beginning of treatments in efficacy studies, the tumor growth kinetics was first assessed for 2 K18GT121 tg/+/p53Δ/Δ/Brca1Δ/Δ tumor lines (A, B) by tumor volume measurements over the period of time. The optimal starting tumor volume was determined to be 0.07–0.2 cm3, which would allow for at let 2 weeks of treatment before the mice had to be euthanized due to tumor burden and/or presence of ascites. C, Kinetics of the tumor development in K18GT121 tg/+/p53Δ/Δ/Brca1Δ/Δ and K18GT121 tg/+/p53Δ/Δ tumor lines indicates delayed start for tumors wild type for Brca1 but very similar growth rates once the tumors are established. (TIF) [file pone.0095649.s003.tif]

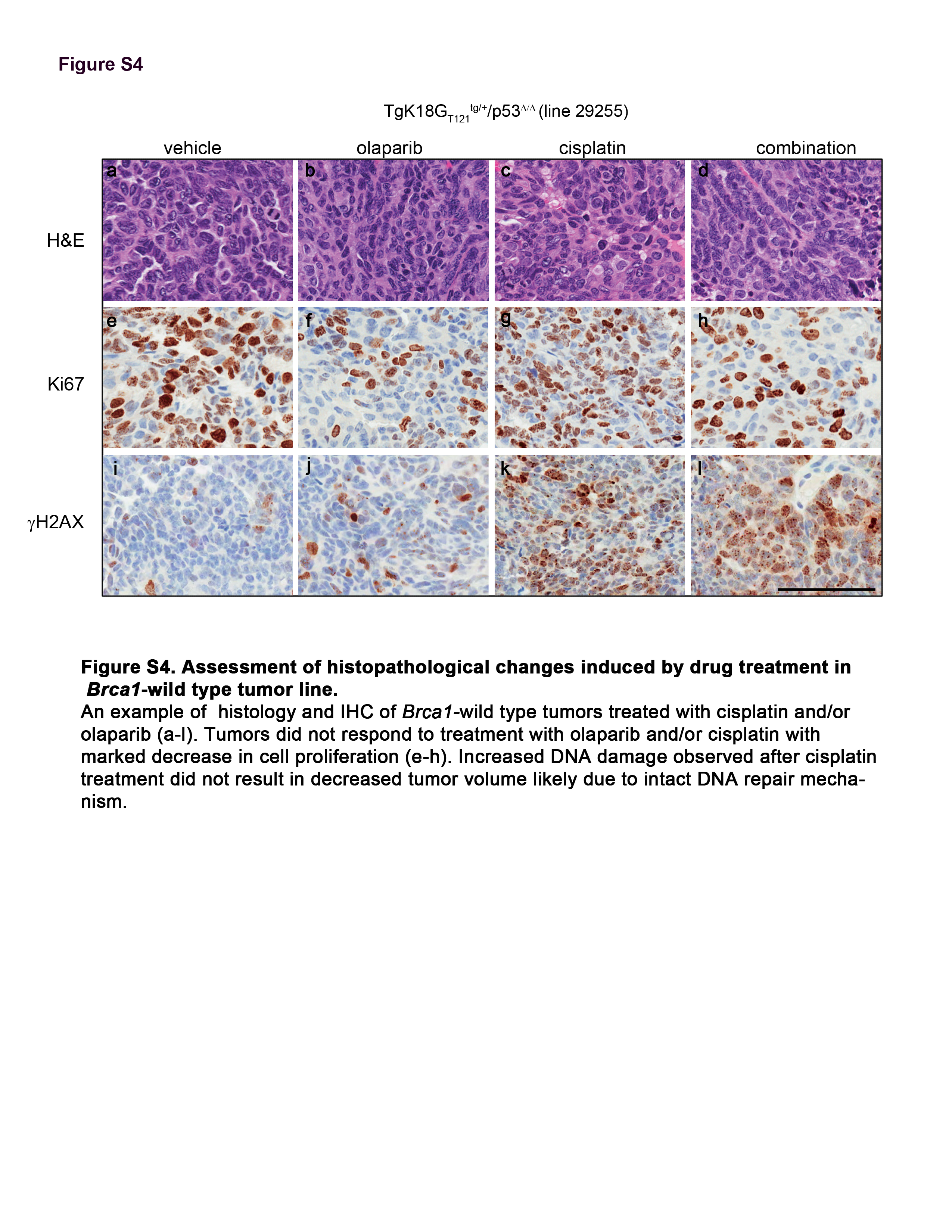

Supplement: Figure S4 — Assessment of histopathological changes induced by drug treatment in Brca1-wild type tumor line. An example of histology and IHC of Brca1-wild type tumors treated with cisplatin and/or olaparib (a–l). Tumors did not respond to treatment with olaparib and/or cisplatin with marked decrease in cell proliferation (e–h). Increased DNA damage observed after cisplatin treatment did not result in decreased tumor volume likely due to intact DNA repair mechanism. (TIF) [file pone.0095649.s004.tif]

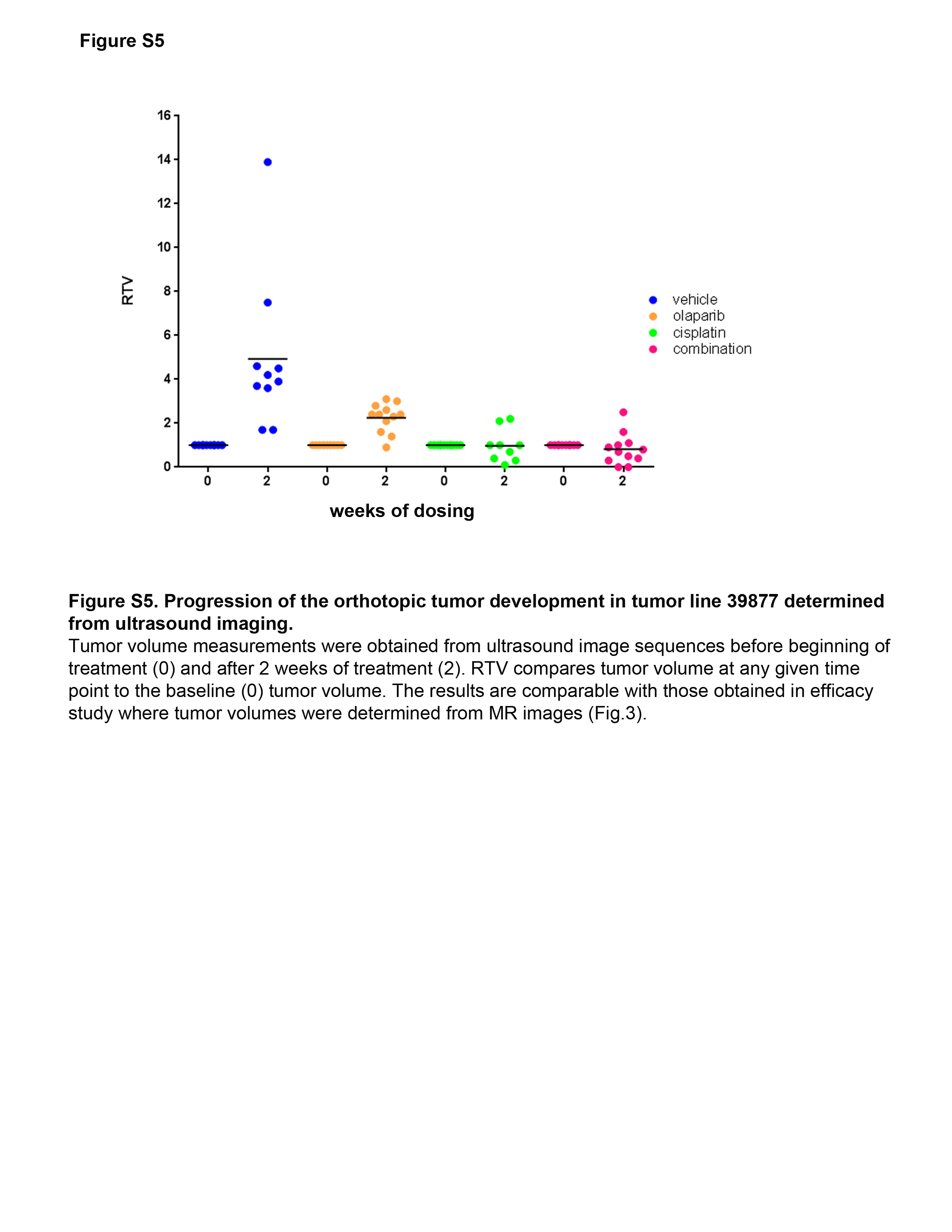

Supplement: Figure S5 — Progression of the orthotopic tumor development in tumor line 39877 determined from ultrasound imaging. Tumor volume measurements were obtained from ultrasound image sequences before beginning of treatment (0) and after 2 weeks of treatment (2). RTV compares tumor volume at any given time point to the baseline (0) tumor volume. The results are comparable with those obtained in efficacy study where tumor volumes were determined from MR images (Fig. 3). (TIF) [file pone.0095649.s005.tif]

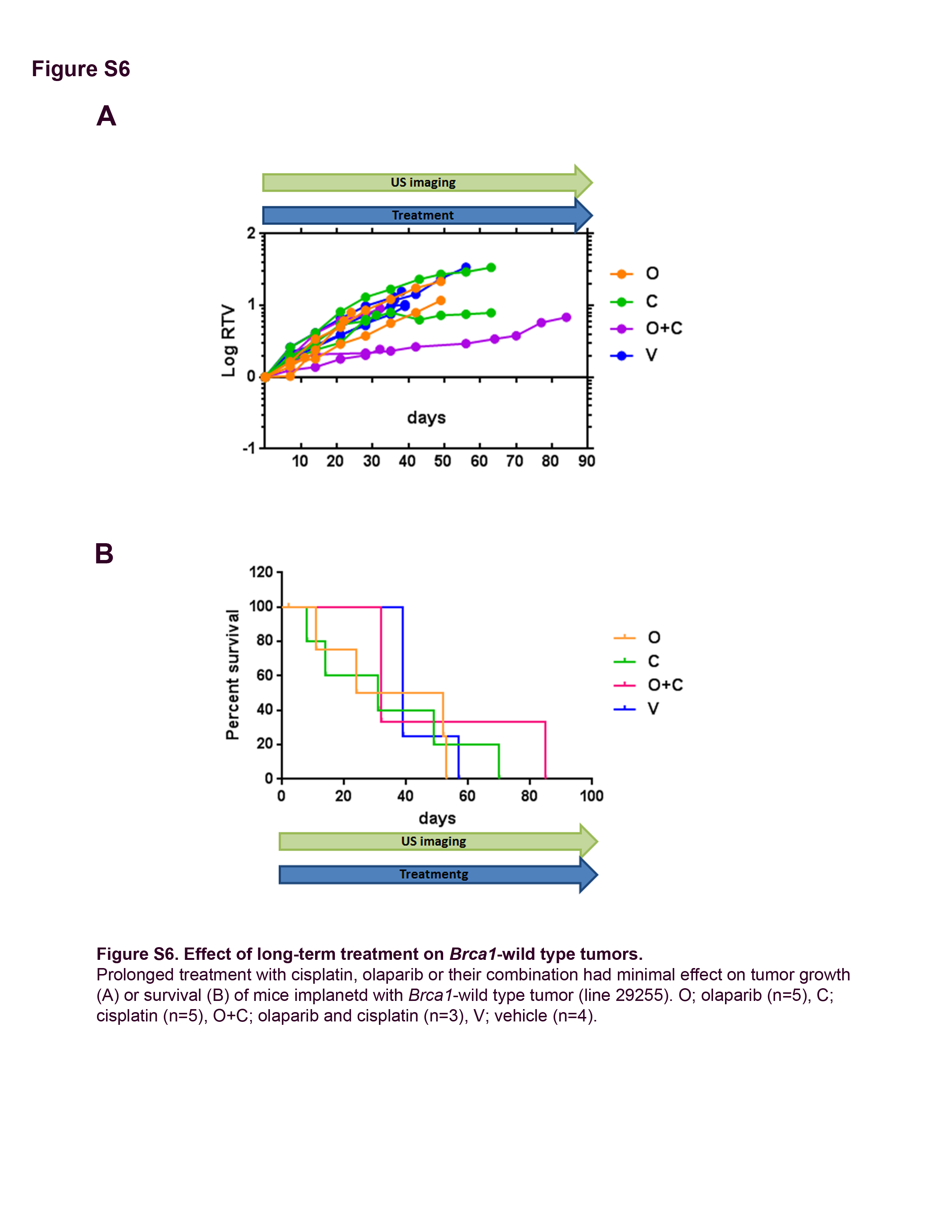

Supplement: Figure S6 — Effect of long-term treatment on Brca1-wild type tumors. Prolonged treatment with cisplatin, olaparib or their combination had minimal effect on tumor growth (A) or survival (B) of mice implanetd with Brca1-wild type tumor (line 29255). O; olaparib (n = 5), C; cisplatin (n = 5), O+C; olaparib and cisplatin (n = 3), V; vehicle (n = 4). (TIF) [file pone.0095649.s006.tif]
