# Supplementary material for: Pathway-Specific Engineered Mouse Allograft Models Functionally Recapitulate Human Serous Epithelial Ovarian Cancer
Source: PLoS One. 2014 Apr 18;9(4):e95649. doi: 10.1371/journal.pone.0095649 (PMC3991711; doi:10.1371/journal.pone.0095649)
Supplement: Table S1 — Summary of the results obtained from orthotopic grafting of cultured cell lines. (DOCX) [file pone.0095649.s007.docx]

**Table S1. Summary of the results obtained from orthotopic grafting of cultured cell lines.**

|  |  | **IMMUNOCOMPROMISED/ I.B. INJECTION** | | | **IMMUNOCOMPROMISED/ I.P. INJECTION** | | | **IMMUNECOMPETENT/ I.B. INJECTION** | | | **IMMUNECOMPETENT/ I.P. INJECTION** | | |
| --- | --- | --- | --- | --- | --- | --- | --- | --- | --- | --- | --- | --- | --- |
| **CELL LINE** | **GENOTYPE** | **TAKE RATE (N)** | **AVERGE LATENCY (MONTHS)** | **INTER-TUMOR HISTOLOGY** | **TAKE RATE (N)** | **AVERGE LATENCY (MONTHS)** | **INTER-TUMOR HISTOLOGY** | **TAKE RATE (N)** | **AVERGE LATENCY (MONTHS)** | **TUMOR HISTOLOGY** | **TAKE RATE (N)** | **AVERGE LATENCY (MONTHS)** | **INTER-TUMOR HISTOLOGY** |
| 22864 TUM | TgK18G_T121_^tg/+^*/*Brca1^Δ/Δ^/p53^R172H/Δ^ | 0% (0/5) | N/A | N/A | 0% (0/5) | N/A | N/A | 0% (0/5) | N/A | N/A | 20% (1/5) | 7.1 | 100% SEOC, papillary |
| 23615 TUM | TgK18G_T121_^tg/+^*/*Brca1^Δ/Δ^/p53^R172H/Δ^ | 100% (5/5) | 5.2 | 80% Undifferentiated carcinoma, 20% SEOC, poorly differentiated papillary | 100% (4/4) | 2.8 | 100% Undifferentiated carcinoma | 20% (1/5) | 4.5 | 100% Undifferentiated carcinoma | 0% (0/4) | N/A | N/A |
| 26341 TUM | TgK18G_T121_^tg/+^*/*Brca1^Δ/Δ^/p53^R172H/Δ^ | 0% (0/5) | N/A | N/A | 0% (0/6) | N/A | N/A | 0% (0/5) | N/A | N/A | 0% (0/5) | N/A | N/A |
| 15825 TUM | TgK18G_T121_^tg/+^*/*Brca2^Δ/Δ^/p53^R172H/Δ^ | 83% (10/12) | 4.5 | 80% SEOC, papillary, 20% SEOC, poorly differentiated papillary | 100% (2/2) | 3.4 | 100% SEOC, poorly differentiated papillary | 0% (0/14) | N/A | N/A | 60% (3/5) | 3.4 | 67% SEOC papillary, 33% undifferentiated carcinoma |
| 23185 TUM | TgK18G_T121_^tg/+^*/*Brca2^Δ/Δ^/p53^R172H/Δ^ | 40% (2/5) | 6.7 | 100% SEOC, papillary | 50% (2/4) | 5.5 | 50% SEOC, papillary, 50% SEOC, poorly differentiated papillary | 0% (0/4) | N/A | N/A | 50% (2/4) | 5.7 | 100% SEOC, papillary |
| 25604 TUM | TgK18G_T121_^tg/+^*/*Brca2^Δ/Δ^/p53^R172H/Δ^ | 20% (1/5) | 7.3 | 100% SEOC, papillary | 0% (0/4) | N/A | N/A | 0% (0/4) | N/A | N/A | 0% (0/5) | N/A | N/A |
| 29410 TUM | TgK18G_T121_^tg/+^*/*Brca2^Δ/Δ^/p53^R172H/Δ^ | 0% (0/5) | N/A | N/A | 0% (0/4) | N/A | N/A | 0% (0/5) | N/A | N/A | 0% (0/5) | N/A | N/A |
| 30200 TUM | TgK18G_T121_^tg/+^*/*Brca1^Δ/Δ^/p53^Δ/Δ^ | 0% (0/5) | N/A | N/A | 75% (3/4) | 3.8 | 67% Undifferentiated carcinoma, 33% SEOC, poorly differentiated papillary | 0% (0/5) | N/A | N/A | 60% (3/5) | 5 | 67% SEOC papillary, 33% undifferentiated carcinoma |
| R5814 TUM | TgK18G_T121_^tg/+^*/*Brca1^Δ/Δ^/p53^Δ/Δ^ | 0% (0/5) | N/A | N/A | 0% (0/5) | N/A | N/A | 0% (0/5) | N/A | N/A | 0% (0/5) | N/A | N/A |
| R5828 TUM | TgK18G_T121_^tg/+^*/*Brca1^Δ/Δ^/p53^Δ/Δ^ | 0% (0/5) | N/A | N/A | 0% (0/5) | N/A | N/A | 0% (0/5) | N/A | N/A | 0% (0/5) | N/A | N/A |
| R5830 TUM | TgK18G_T121_^tg/+^*/*Brca1^Δ/Δ^/p53^Δ/Δ^ | 0% (0/6) | N/A | N/A | 0% (0/5) | N/A | N/A | 0% (0/5) | N/A | N/A | 0% (0/5) | N/A | N/A |
| R5831 TUM | TgK18G_T121_^tg/+^*/*Brca1^Δ/Δ^/p53^Δ/Δ^ | 0% (0/5) | N/A | N/A | 0% (0/4) | N/A | N/A | 0% (0/5) | N/A | N/A | 0% (0/5) | N/A | N/A |
| R5843 TUM | TgK18G_T121_^tg/+^*/*Brca1^Δ/Δ^/p53^Δ/Δ^ | 0% (0/5) | N/A | N/A | 0% (0/5) | N/A | N/A | 0% (0/5) | N/A | N/A | 0% (0/5) | N/A | N/A |
| R5848 TUM | TgK18G_T121_^tg/+^*/*Brca1^Δ/Δ^/p53^Δ/Δ^ | 0% (0/5) | N/A | N/A | N/A | N/A | N/A | 0% (0/5) | N/A | N/A | 0% (0/4) | N/A | N/A |
| R5860 TUM | TgK18G_T121_^tg/+^*/*Brca1^Δ/Δ^/p53^Δ/Δ^ | 100% (5/5) | 4.8 | 80% SEOC, papillary, 20% SEOC, poorly differentiated papillary | 80% (4/5) | 5.4 | 50% SEOC, papillary, 50% Undifferentiated carcinoma | 0% (0/5) | N/A | N/A | 0% (0/5) | N/A | N/A |
| R5836 TUM | TgK18G_T121_^tg/+^*/*p53^Δ/Δ^ | 75% (3/4) | 5.8 | 100% SEOC, papillary | N/A | N/A | N/A | 0% (0/5) | N/A | N/A | 77% (7/9) | 4.9 | 72% SEOC, poorly differentiated papillary, 14% SEOC, papillary, 14% undifferentiated carcinoma |
| 26341 ASC | TgK18G_T121_^tg/+^*/*Brca1^Δ/Δ^/p53^R172H/Δ^ | 25% (1/4) | 3.1 | 100% SEOC, papillary | 20% (1/5) | 4.4 | 100% Undifferentiated carcinoma | 0% (0/5) | N/A | N/A | 0% (0/5) | N/A | N/A |
| 23615 ASC | TgK18G_T121_^tg/+^*/*Brca1^Δ/Δ^/p53^R172H/Δ^ | 30% (3/10) | 5.8 | 100% sarcoma | 10% (1/10) | 6.2 | 100% Undifferentiated carcinoma | 0% (0/9) | N/A | N/A | 0% (0/4) | N/A | N/A |
| 24661 ASC | TgK18G_T121_^tg/+^*/*Brca1^Δ/Δ^/p53^R172H/Δ^ | 0% (0/5) | N/A | N/A | 0% (0/4) | N/A | N/A | 0% (0/5) | N/A | N/A | 0% (0/5) | N/A | N/A |
| 15825 ASC | TgK18G_T121_^tg/+^*/*Brca2^Δ/Δ^/p53^R172H/Δ^ | 30% (3/10) | 6.3 | 100% SEOC, papillary | 0% (0/5) | N/A | N/A | 0% (0/7) | N/A | N/A | 66% (2/3) | 6.85 | 100% Undifferentiated carcinoma |
| 23172 ASC | TgK18G_T121_^tg/+^*/*Brca2^Δ/Δ^/p53^R172H/Δ^ | 0% (0/5) | N/A | N/A | 0% (0/4) | N/A | N/A | 0% (0/5) | N/A | N/A | 0% (0/5) | N/A | N/A |
| R5854 ASC | TgK18G_T121_^tg/+^*/*Brca1^Δ/Δ^/p53^Δ/Δ^ | 33% (2/6) | 7.7 | 50% SEOC, papillary, 50% undifferentiated carcinoma | N/A | N/A | N/A | 0% (0/5) | N/A | N/A | N/A | N/A | N/A |

*latency of tumor development from cell injections till end point

*TgK18G_T121_^tg/+^ =* transgenic for bacterial artificial chromosome containing the mouse cytokeratin 18 gene, into which a Cre-conditional loxP-GFP-stop-loxP T_121_ cassette was inserted

*Brca1^Δ/Δ^ =* deletion mutant for *Brca1* gene

*Brca2^Δ/Δ^ =* deletion mutant for *Brca2* gene

*p53^Δ/Δ^* = deletion mutant for p53 gene

*p53^R172H/Δ^* = point mutation and deletion mutant for *p53* gene

TUM = primary tumor-derived cell line

ASC = ascites derived-cell line
