# Supplementary material for: Pathway-Specific Engineered Mouse Allograft Models Functionally Recapitulate Human Serous Epithelial Ovarian Cancer
Source: PLoS One. 2014 Apr 18;9(4):e95649. doi: 10.1371/journal.pone.0095649 (PMC3991711; doi:10.1371/journal.pone.0095649)
Supplement: Data S1 — Supplementary Data contain material and methods for establishment of murine primary ovarian cancer cell lines, methods and results for cell implantations, methods for imaging and tumor volume measurements, tissue collection, pathological and IHC analysis, quantitative analysis of IHC stains, immunofluorescent staining of human and murine cells and quantitative PCR analysis of Brca1 status. (DOCX) [file pone.0095649.s008.docx]

**Supplementary Data**

**Pathway-specific engineered mouse allograft models functionally recapitulate human serous epithelial ovarian cancer**

**Establishment of primary tumor lines from the SEOC GEM model**

All murine ovarian carcinoma cell lines were established in accordance to animal study protocol approved by the Institutional Animal Care and Use Committee of Frederick National Laboratory for Cancer Research. Serous epithelial ovarian tumors generated in GEM models [[1](#_ENREF_1)] were used to prepare primary tumor cell cultures (Table S1). Briefly, tumor fragments were washed in sterile PBS containing penicillin/streptomycin (Life Technologies, Grand Island, NY), minced and dissociated by 10mg/ml collagenase/dispase (Roche, Indianapolis, IN) treatment at 37°C for 1 hour under constant mixing. The cell suspension was filtered through a cell strainer (BD Biosciences, Chicago, IL) to remove any undigested tissue residues, cells were spun, washed twice with fresh media [[2](#_ENREF_2)] and plated on collagen coated plates. To establish cell cultures from ascites, ascites were spun and cells were washed several times with PBS and plated on collagen coated dishes in complete media. Once the cells attached to the plates (about 2 days later), the cells were washed several times with PBS to remove any residual blood cells and debris and cultured in fresh media. Cultured mouse primary ovarian cancer cells (p4-p14) were washed with PBS and harvested by 0.25% Trypsin-EDTA, washed several times in PBS, counted and re-suspended in 4% methylcellulose (10^6^cells/5µl) for intrabursal injection (i.b.) or in PBS (10^7^cells/200µl) for intraperitoneal injection (i.p.) and kept on ice until time of injection.

**Cell implantations**

To perform orthotopic cell injection the animals were prepared for surgical procedure as described above. Hamilton syringe with 32G custom made needle was used to inject 5 µl of cell suspension under the bursa of the exposed ovary. The ovaries were replaced back into the abdominal cavity, the peritoneum was sutured and the skin closed with metal clips.

Several of the cultured lines were tested for tumorigenic potential following intraperitoneal (i.p.) and intrabursal (i.b.) introduction into both immunocompromised and immunocompetent recipient mice (Table S1). This approach proved much less reliable in establishing tumors than tissue transplantation: 37% (6/16) and 31% (5/16) of primary tumor-derived cell lines and 66% (4/6) and 16% (1/6) of ascites-derived cell lines re-established ovarian tumors and disseminated disease with SEOC-like characteristics or undifferentiated carcinomas upon i.b. or i.p. injection, respectively, in immunocompromised recipients (Table S1). However, only 1 of 22 cell lines injected under the bursa of immunocompetent recipients re-established tumors (1 mouse out of 5 injected). 31% (5/16) of primary tumor-derived and one of 6 ascites-derived cell lines successfully engrafted upon i.p. injection into immunocompetent mice (Table S1). The take-rate was variable in different cell lines and morphology of tumors resulting from cell injections often varied even within the same cell line. 44% of all tumors resulting from any type of cell injection had SEOC with papillary histology, 23% had SEOC with poorly differentiated papillary histology and 33% undifferentiated carcinoma (detailed in Table S1).

**Tissue collection, pathological and IHC analysis**

Animals were euthanized by CO_2_ inhalation, organs were fixed in 10% neutral buffered formalin, processed for paraffin embedding and characterized by microscopic evaluation. Five µm serial sections were cut for H&E and IHC. Pathological evaluation of histological findings was performed by a board certified veterinary pathologist (P.L.M.).

**Histopathological criteria for defining the tumor differentiation**

Histopathological criteria for defining the differentiation were as follows: tumors designated as SEOC papillary were well differentiated, with the majority of the tumor section being comprised of well differentiated papillary structures with occasional glandular or micro-cystic profiles, occasional poorly differentiated foci were present. Tumors with a majority of the section lacking well differentiated papillary structures were designated as SEOC poorly differentiated papillary. Large portions of these tumors consisted of solid sheets with occasional tightly compressed papillary structures; however, these tumors always contained a significant, yet minor, percentage of papillary structures. Tumors lacking characteristic histopathological pattern and significant regions that were suggestive of any specific epithelial cell type or tissue of origin were designated as Undifferentiated carcinoma. Rare foci of papillary or glandular histology may have been present in these tumors but represented less than 1% of the tumor section area. Paraffin sections were deparaffinized in xylene, rehydrated in ethanol according to standard protocol and subsequently used for IHC stains. IHC for Ki67 was performed as published previously [[1](#_ENREF_1)], the same protocol was used for IHC for γH2AX with rabbit monoclonal antibody (Cell Signaling, Danvers, MA).

**Imaging and tumor volume measurements**

Magnetic resonance imaging (MRI) was performed as previously described [[1](#_ENREF_1)]. Baseline and terminal tumor volumes were measured from MR image sequences using Image J or MIM 5.2.2 software. For ultrasound imaging (US), the abdominal area of mice was shaved prior to imaging, followed by fine hair removal using Surgi-cream (Surgi-Care, Waltham, Massachusetts). A high frequency (40 MHz) Ultrasound imaging system (Vevo 2100, VisualSonics, Toronto, Canada), was used in these studies. 3D B-mode images were acquired using the MS-550S or for larger volumes the MS250 transducer: axial resolution of 40µm and 75µm, respectively, and a lateral spatial resolution of 80µm and 165µm, respectively. The acoustic focus was placed at the center of the ovary. Throughout the imaging session, mice were kept anesthetized with 1-2% isoflurane in oxygen at 1L/min on a heated stage according to the manufacturer’s protocol. Respiratory gating was used to synchronize data acquisition with the mouse respiratory cycle to reduce motion artifact during image analysis. Ovarian volumes were analyzed using the vendor analysis software (VisualSonics, Toronto, Canada).

**Quantitative analysis of IHC stains**

The ARIOL SL-50 Automated Slide Scanner (Leica Microsystems, Buffalo Grove, IL) was used to quantify both proliferation (Ki-67) and DNA damage (γH2AX) on 20X scans. For Ki-67 the *kisight* analysis module was used for automated counting of DAB positive (brown) nuclei and hematoxylin stained (blue) negative nuclei. For γH2AX the *kisight* module was used to assess the percentage of nuclear area positive for γH2AX. Total area of pixels positive for γH2AX (brown) was divided by the total area of pixels in nuclei in the tumor section. Regions of necrosis were excluded from all analyses.

**Quantitative PCR analysis of *Brca1* status**

Relative content of genomic *Brca1* in tumor tissue or cultured cells was examined by qPCR amplifying a target located between two loxP sites of the floxed *Brca1* allele. DNA from cultured cells or frozen tumors was extracted by DNeasy Blood and Tissue Kit (Qiagen, Valencia, CA) and 20 ng of DNA was used in qPCR reaction using SYBR Green PCR Master Mix (Applied Biosystems, Grand Island, NY) and 500 pmol of each primer (Brca1 ex11 Fw: 5’-GGAAGGGTAGCAGCAGTGAC-3’, Brca1 ex11Rev: 5’-GGGAGTTTGCATTTGCAGTT-3’). Amplification of endogenous control beta-actin was performed for each sample using primers: beta-actin ex4 Fw: 5’-AGCCATGTACGTAGCCATCC-3’, beta-actin ex4 Rev: 5’-TTTGATGTCACGCACGATTT-3’. All samples were run in triplicates. Comparative Ct method was used to evaluate the relative quantity of *Brca1* gene using formula 2^-ΔCt^ where ΔCt is (mean Ct_Brca1_ – mean Ct_actin_). Content of *Brca1* was plotted relative to the average genomic *Brca1* content in wild type tumors or cells.

**Phosphohistone H2A.X and Rad 51 immunofluorescence**

Human or murine ovarian carcinoma cells were seeded on coverslips and grown for 24 hrs before being irradiated with 10 Gy. Six hours after irradiation cells were fixed in 2% paraformaldehyde in PBS and permeabilized in 0.2% Triton X-100 in PBS for 20 min. Blocking (30 min) and staining were performed in 1% BSA, 0.15% glycine, and 0.1% Triton X-100 buffer at room temperature. All washing steps were done in PBS with 0.1% Triton X-100. Incubations with phosphohistone H2AX rabbit monoclonal (Cell Signaling, Danvers, MA) and Rad 51 rabbit polyclonal antibodies (Santa Cruz, TX) were done for 2 hours. Alexa Fluor 546 donkey anti-rabbit IgG (Invitrogen, Grand Island, NY) was used as secondary antibody and ProLong Gold antifade reagent with DAPI (Invitrogen, Grand Island, NY) was used to mount coverslips. Images were taken with Zeiss Axio Imager.M1 microscope using 40 x objective equipped with AxioCam HR Rev3 camera. Images were acquired and processed by AxioVision 4.8.2 software through multidimensional acquisition.

**References**

1. Szabova L, Yin C, Bupp S, Guerin TM, Schlomer JJ, Householder DB, Baran ML, Yi M, Song Y, Sun W, McDunn JE, Martin PL, Van Dyke T, Difilippantonio S. (2012) Perturbation of Rb, p53 and Brca1 or Brca2 cooperate in inducing metastatic serous epithelial ovarian cancer. Cancer Res 72: 4141-4153.
2. Roby KF, Taylor CC, Sweetwood JP, Cheng Y, Pace JL, et al. (2000) Development of a syngeneic mouse model for events related to ovarian cancer. Carcinogenesis 21: 585-591.
